# High-flow nasal cannula versus noninvasive ventilation in stabilized hypercapnic exacerbation: a physiological crossover trial

**DOI:** 10.1016/j.aicoj.2026.100092

**Published:** 2026-05-19

**Authors:** Fernando Vieira, Annia Schreiber, Mattia Docci, Antenor Rodrigues, Vorakamol Phoophiboon, Carles Subira, Matthew Ko, Mayson L.A. Sousa, Tai Pham, Thomas Piraino, Remi Coudroy, Giulia Cavalot, Irene Telias, Detajin Junhasavasdikul, Martin Dres, Michael C. Sklar, Laurent Brochard

**Affiliations:** a Keenan Research Centre, Li Ka Shing Knowledge Institute, St. Michael's Hospital, Unity Health Toronto, ON, Canada; b Interdepartmental Division of Critical Care Medicine, University of Toronto, Toronto, ON, Canada; c Fisher & Paykel Healthcare Ltd., Auckland, New Zealand; d Division of Critical Care Medicine, Department of Medicine, Faculty of Medicine, Chulalongkorn University, Bangkok, Thailand; e Critical Care Department, Institut de Recerca Sant Pau (IR Sant Pau), Hospital de la Santa Creu i Sant Pau, Barcelona, Spain; f Translational Medicine Program, Research Institute, Hospital for Sick Children, University of Toronto, Toronto, Canada; g Department of Respiratory Therapy, College of Rehabilitation Sciences, Rady Faculty of Health Sciences, University of Manitoba, Winnipeg, Canada; h AP-HP, Hôpital de Bicêtre, DMU CORREVE, Service de Médecine Intensive-Réanimation, FHU SEPSIS, Groupe de Recherche Clinique CARMAS, Université Paris-Saclay, Le Kremlin-Bicêtre, France; i Inserm U1018, Equipe d'Epidémiologie Respiratoire Intégrative, Centre de Recherche en Epidémiologie et Santé des Populations, Université Paris-Saclay, UVSQ, Univ. Paris-Sud, Villejuif, France; j Department of Anesthesia, McMaster University, Hamilton, ON, Canada; k CHU de Poitiers, Service de Médecine Intensive Réanimation, Poitiers, France; INSERM CIC 1402, IS-ALIVE Research Group, Université de Poitiers, Poitiers, France; l Department of Emergency Medicine, San Giovanni Bosco Hospital, Torino, Italy; m Division of Respirology and Critical Care Medicine, University Health Network and Sinai Health System; n Division of Pulmonary and Pulmonary Critical Care, Department of Medicine, Faculty of Medicine Ramathibodi Hospital, Mahidol University, Bangkok, Thailand; o Neurophysiologie Respiratoire Expérimentale et Clinique, INSERM, UMRS1158, Paris, France; p Service de Médecine Intensive - Réanimation (Département "R3S"), Paris, France

**Keywords:** High-flow nasal cannula, Non-invasive ventilation, Diaphragm ultrasound, Hypercapnic respiratory failure, Non-inferiority

## Abstract

**Background:**

Evidence comparing high-flow nasal cannula (HFNC) and non-invasive ventilation (NIV) in acute hypercapnic respiratory failure remains controversial. We compared their short-term effects on breathing effort, ventilation, CO_2_ clearance, and preference.

**Methods:**

A randomized, crossover, non-inferiority trial was conducted in patients with stabilized hypercapnic exacerbation requiring NIV or HFNC. Baseline oxygen therapy was followed by a randomized sequence of NIV and HFNC at 30 and 50 L.min^−1^. The primary endpoint was to assess non-inferiority of HFNC 50 L.min^−1^ compared to NIV. Diaphragm, parasternal intercostal, and transversus abdominis muscle activity were assessed using thickening fraction (TF) and the product of TF and respiratory rate (TF•RR). Ventilation was evaluated using electrical impedance tomography and transcutaneous partial pressure of carbon dioxide (tcCO_2_).

**Results:**

21 patients (mean ± SD age 69 ± 11 years, 82% COPD) were enrolled. In 17, diaphragm thickening fraction (TFdi) was available: HFNC 50 L.min^−1^ was non-inferior to NIV in reducing TFdi (p = 0.122, 95% CI: −19.1–3.4), as was HFNC at 30 L.min^−1^ (p = 0.413, 95% CI: −17.0–5.7). Only HFNC 50 L.min^−1^ reduced TFdi•RR (p = 0.036) and respiratory rate compared to baseline (p = 0.001). HFNC at 50 L.min^−1^ decreased the baseline TFdi by 18% ± 36% (p = 0.033), whereas NIV did not decrease it. HFNC and NIV reduced tcCO₂ compared to baseline. Minute ventilation and the estimated ventilatory ratio were lower with HFNC than NIV (p < 0.01). HFNC was the preferred strategy by the patients.

**Conclusions:**

In stabilized hypercapnic exacerbation, HFNC and NIV reduced tcCO₂, but only HFNC lowered ventilatory ratio and minute ventilation. HFNC at 50 L.min^−1^ reduced diaphragm activity and was non-inferior to NIV in this regard, while being preferred by patients.

## Introduction

Acute hypercapnic respiratory failure occurs in patients with chronic conditions such as chronic obstructive pulmonary disease (COPD), asthma, obesity hypoventilation, congestive heart failure and sleep apnea syndrome [[1], [2], [3], [4]]. These etiologies often overlap under the umbrella of undifferentiated hypercapnic respiratory failure, resulting in similar acute presentations and short-term outcomes, and responding to the same supportive therapies, mostly noninvasive ventilation (NIV) [3,5]. NIV is the standard therapy for COPD exacerbation, unloading inspiratory muscles while providing carbon dioxide (CO_2_) clearance [6,7]. By avoiding intubation and invasive mechanical ventilation, NIV can improve outcomes [[8], [9], [10]], however its failure rate is approximately 15–20% and the mortality associated with failure can be as high as 45% [11]. NIV is not always well tolerated in mild exacerbations, highlighting the need for more comfortable respiratory support strategies.

High-flow nasal cannula (HFNC) offers potential benefits in hypercapnic respiratory failure by providing low levels of positive pressure counteracting intrinsic positive end-expiratory pressure (PEEP), decreasing anatomical dead space through the clearance of expired carbon dioxide (CO_2_) in the upper airways, and enhancing comfort compared to conventional oxygen [[12], [13], [14], [15], [16], [17]]. In COPD exacerbation, HFNC showed a short-term comparable CO_2_ clearance [[18], [19], [20]], better comfort and lower incidence of skin breakdowns than NIV [21]. HFNC seems to be superior to conventional oxygen therapy in acute hypercapnic respiratory failure [22]. However, the current body of evidence arising from randomized trials and summarized in systematic reviews and meta-analysis is insufficient to determine whether HFNC is superior, inferior, or equivalent to NIV for patients with acute hypercapnic respiratory failure due to imprecision and study heterogeneity [23,24].

This underscores the need for a deeper understanding of the physiology behind adopting HFNC or NIV in managing acute hypercapnic respiratory failure. This study aims to compare the short-term physiological effects of HFNC and NIV on inspiratory effort, CO_2_ clearance and comfort in patients with exacerbations. We hypothesized that HFNC at 50 L.min^−1^ (HFNC50) would be non-inferior to NIV in reducing inspiratory effort, assessed by diaphragm thickening fraction (TFdi).

## Methods

More detailed methodological information, including patient enrollment criteria, data collection procedures, ultrasound imaging protocols, EIT calibration, statistical modeling, and sample size justification is available in Supplementary eText-1.

This study was approved by the Unity Health Toronto Research Ethics Board (REB 16-389) and registered on ClinicalTrials.gov (NCT03033251). This physiological investigation was a prospective, randomized, cross-over, non-inferiority trial comparing HFNC therapy at different flow rates (30 & 50 L.min^−1^) and NIV. Informed consent was obtained from the patients or their substitute decision-makers. Enrollment was halted during the COVID-19 pandemic and resumed after the pandemic. In accordance with reporting standards, this study follows the CONSORT 2010 Statement: Extension for Randomized Crossover Trials [25]. A CONSORT checklist is provided in supplemental eTable-1.

### Patients

We included adult patients (>40 years of age to ensure homogeneity in the population) admitted to the Emergency Department, ICU, or medical wards at St. Michael's Hospital, in Toronto, Canada. The investigation initially enrolled only patients with exacerbated COPD (6 patients enrolled in this phase) but was later amended (March 2023) to include those with undifferentiated acute on chronic hypercapnic respiratory failure requiring non-invasive respiratory support (NIV or HFNC) because of the difficulty to have a precise diagnosis on admission. Eligibility criteria included: respiratory acidosis (defined as arterial pH ≤ 7.35 and PCO_2_ ≥45 mmHg; or venous pH ≤ 7.34 and PvCO_2_ ≥50 mmHg), a respiratory rate (RR) ≥20 breaths/min, and the ability to tolerate spontaneous breathing with conventional oxygen therapy for 15 min (if deemed safe by the attending clinician).

Patients with urgent need for intubation, or morbid obesity (Body Mass Index - BMI >40 kg/m^2^) were excluded as this latter condition could compromise accurate ultrasound and electrical impedance tomography (EIT) assessments. Patients with severe respiratory acidosis (arterial pH < 7.25 or venous pH < 7.20), a decreased level of consciousness (GCS ≤ 11), hemodynamic instability needing vasopressors, bronchopleural fistula, or uncooperative behavior were initially excluded but could be included if improved within the subsequent 72 h.

### Study protocol

Measurements were performed during conventional oxygen therapy (COT) as baseline, followed by a randomized sequence of NIV, and HFNC at two flow rates (30 & 50 L.min^−1^) each administered for 15 min, similar to a previous study [26] in a standardized clinical setting, ensuring consistency in timing and monitoring across conditions. We performed a two-step randomization using sealed opaque envelopes, first we randomized the therapy (HFNC or NIV), followed by the order of HFNC flow rates (30 L.min^−1^ or 50 L.min^−1^), see eFigure-1. A 5-minute washout period with COT (via nasal cannula or Venturi mask titrated to maintain SpO₂ within the range specified by the attending physician) was included between NIV and HFNC, as well as between the HFNC flow rates. This short duration, which helped to reduce the total duration of the protocol, was considered sufficient to minimize carry-over effects since only the last 5 min of each condition were analyzed. During the final 5 min of each condition, vital signs (i.e., blood pressure, heart rate, and respiratory rate) were collected, and ultrasound images of the respiratory muscles were acquired.

HFNC therapy was delivered using the Bellavista-1000e ventilator (Vyaire Medical Inc, Mettawa, IL, USA), with gas heated and humidified (M850, Fisher & Paykel Healthcare, Auckland, NZ) to 37 °C (or 31 °C if uncomfortable) delivered in accordance with standard practice at the time of protocol conception, using symmetrical, medium-sized nasal cannulas (OPT944, Fisher & Paykel Healthcare) at the baseline FiO_2_.

NIV was also delivered using the Bellavista-1000e ventilator in a dual-limb configuration through a non-vented oronasal mask (FreeMotion RT041, Fisher & Paykel Healthcare, Auckland, NZ). For all patients, the ventilator settings were initiated according to standard local practice at St. Michael's Hospital for hypercapnic patients initiated on NIV, which consists of starting with a PEEP of 5 cmH_2_O and a pressure support of 8–10 cmH_2_O, with subsequent adjustments made according to clinical judgment (Table 2). For patients already receiving NIV prior to the study, their settings were maintained unchanged throughout.

**Table 2:** Physiological and Clinical Variables at baseline and under different respiratory support modalities.

		HFNC			
Variables	Baseline COT	30 L.min^−1^	50 L.min^−1^	NIV	p-value
TFdi, %	23.1 (19.4–47.9)	27.3 (16.4–34.9)	22.9 (16.7–31.2)	26.0 (20.1–49.8)	0.113
TFdi vs baseline (%change)	Ref.	−7.5 ± 42	−17.7 ± 36†	30.1 ± 85	0.031
RR, breaths/min	22 (21–52)	22 (19–25)	20 (17–25)*	21 (18–24)	0.003
TFdi • RR product	5.5 (4.3–12.0)	5.8 (4.6–7.5)	4.4 (3.7–7.0)*	7.0 (4.1–9.6)	0.024
TFdi • RR vs baseline (%change)	Ref.	−13.5 ± 37.2	−27.0 ± 34.8†	25.1 ± 76.9	0.013
TFpi, %	10.6 (5.65–18.5)	9.8 (7.1–13.3)	12.3 (8.8–18.1)	13.0 (5.2–17.9)	0.388
TFpi•RR Product	2.7 (1.2–3.9)	2.4 (1.7–2.6)	2.4 (1.8–3.9)	3.0 (1.2–3.2)	0.554
TFtra, %	18.3 (12.3–25.9)	18.5 (10.3–25.3)	20 (10.6–23.7)	14.5 (11.1–20.7)	0.783
Heart rate, bpm	94 ± 13	92 ± 13	96 ± 18	92 ± 14	0.150
SBP, mmHg	129 ± 20	125 ± 22	130 ± 24	129 ± 19	0.221
DBP, mmHg	69 ± 13	69 ± 12	72 ± 15	73 ± 15	0.060
SpO_2_, %	95 ± 4	94 ± 3	95 ± 3	95 ± 3	0.052
SpO_2_/FiO_2_, ratio	323 ± 76	320 ± 78	323 ± 80	324 ± 77	0.088
Tidal Volume, mL	404 (296–595)	434 (316–549)	422 (317–558)	525 (395–604)	0.074
Changes in EELV** vs baseline (mLchange)	Ref.	+53 ± 288†	+139 ± 327†	+443 ± 426	0.003
Absolute V-D difference** (Δ%)	22.3 ± 14.2	26.3 ± 16.4	27.7 ± 15.7	28.5 ± 16.9	0.457
Dyspnea, 0−10	3 (2–3)	2 (0–4)	1 (1–3)	2 (1–4)	0.072
PEEP/PS median(IQR), cmH_2_O	–	–	–	6 (5−6)/7 (6−9)	

Abbreviations: COT = Conventional Oxygen Therapy; HFNC= High Flow Nasal Cannula; NIV = Non-invasive Ventilation, RR = Respiratory Rate; TFdi = Thickening Fraction of the diaphragm; TFpi = Thickening Fraction of the parasternal intercostal; TFtra = Thickening Fraction of the transversus abdominis; SBP = systolic blood pressure; DBP = diastolic blood pressure, SpO_2_ = peripheral oxygen saturation; EELV = End-expiratory Lung Volume, Absolute V-D difference = Absolute ventral‑to‑dorsal ventilation difference, as the absolute difference between ventral and dorsal layers (%), expressed as positive values regardless of dominance; PEEP = End-Expiratory positive airway pressure, PS = Pressure Support, IQR = Interquartile range.

* = Significant post hoc test compared to Baseline, p-value < 0.05.

** = Only 15 patients, who were assessed using EIT.

† = Significant post hoc test compared to NIV, p-value < 0.05.

### Ultrasound

We measured diaphragm thickening fraction (TFdi) as a surrogate of the inspiratory effort [27], and to estimate of the patient’s power of breathing we calculated the TFdi-respiratory rate product (TFdi•RR). Ultrasound images for thickening fraction of the right diaphragm, parasternal intercostal (TFti) and transversus abdominis (TFtra) muscles were acquired using a 12−15 MHz linear array transducer (Ultrasound System Vivid e95, General Electric Healthcare, Boston, MA, USA) by one of the two operators, (AS and GC with 10, and 3 years of expertise in the technique, respectively).

Thickening fraction (TF) within the last 5 min of each condition was calculated as the percentage change in muscle thickness between end-expiration and peak inspiration for the inspiratory muscles, and between end-inspiration and peak expiration for the transversus abdominis, reflecting in both cases the percentage change between the most relaxed condition and peak contractile effort of the respective muscle, averaged over 3–5 breaths.

Obtaining reliable images (and therefore accurate measurements) of the diaphragm was not possible in 3 patients due to difficult visualization or discrimination of the two layers defining the muscle boundaries, related to extreme diaphragmatic thinning or to poor acoustic window due to hyperinflation.

### Electrical Impedance Tomography (EIT)

Two noninvasive impedance-based techniques, calibrated against the ventilator, were used to measure tidal volume, respiratory rate and minute ventilation (MV), the ExSpiron (ExSpiron 1Xi, Respiratory Motion, Inc., Waltham, MA, USA) in 6 initial patients, and the EIT (PulmoVista 500, Dräger, Lübeck, Germany) in 15 following patients. The EIT also was used to monitor changes in end-expiratory lung volume (EELV) and ventral-to-dorsal ventilation difference (V-D difference) [28]. We calibrated EIT against the tidal volume displayed by the ventilator during NIV, using a series of 20 consecutive breaths. During this process, EIT data were recorded at 50 Hz, alongside the tidal volume of each corresponding breath. Volume tracing enabled the computation of tidal volume, respiratory rate, and MV at each study step.

### Transcutaneous CO_2_

A transcutaneous CO_2_ (tcCO_2_) sensor probe (SenTec Digital Monitoring System - SenTec, Therwil, Switzerland), was attached after appropriate calibration via an ear clip to the patients’ earlobe and data were recorded at 0.25 Hz, to non-invasively estimate arterial CO_2_ levels continuously throughout the study protocol.

Ventilatory ratio was estimated non-invasively by integrating ventilatory parameters from processed EIT data (MV), tcCO_2_, and demographic data. This approach utilizes the classical formula used with invasive methods (mechanical ventilation data and arterial blood gas): [MV (mL. min^−1^) × PaCO_2_ (mmHg)]/[predicted body weight × 100 (mL. min^−1^) × 37.5 (mmHg)] [29]. The ventilatory ratio is an indicator of ventilatory inefficiency and dead space.

### Dyspnea and preference

At the end of each condition, we asked the patient to subjectively rate their level of dyspnea (on a 0−10 scale, where 0 = extremely easy to breathe and 10 = extremely difficult to breathe) [30]. At the end of the protocol, we asked patients to indicate their preferred device (in terms of comfort) among COT, HFNC, and NIV, as well as their preference between HFNC and NIV, and their preferred HFNC flow rate.

## Endpoints

In this cross-over non-inferiority study, the primary endpoint was the absolute diaphragm thickening fraction per breath, looking for a non-inferiority of HFNC50 compared to NIV. Secondary endpoints included comparisons between NIV and HFNC30, as well as between difference between HFNC flow rates. Additional outcomes assessed were MV, RR, CO_2_ clearance, a surrogate of patient’s power of breathing (TFdi•RR product), TF of parasternal intercostal and transversus abdominis, dyspnea, and preference.

## Statistical analysis

After assessing normality using the Shapiro–Wilk test, continuous variables were presented as mean and standard deviation (SD) for normally distributed data, or as median and interquartile range (IQR) for non-normally distributed data. Categorical variables were expressed as frequencies and percentages. Non-inferiority test between HFNC50 and NIV was evaluated using the Wilcoxon signed-rank test with Hodges–Lehmann estimates and 95% confidence intervals, applying a non-inferiority margin of absolute 10 percentage points. A 10% non-inferiority margin was chosen to reflect a difference considered clinically acceptable, supported by methodological considerations.

An exploratory non-inferiority analysis was performed comparing HFNC30 with NIV. In addition, a 10% relative change from baseline in TFdi was used as an exploratory non-inferiority margin to compare both HFNC30 and HFNC50 against NIV. As a sensitivity analysis, a subgroup non-inferiority test was conducted between HFNC50 and NIV, stratifying patients by the pH value closest to the time of data recording. Patients were divided into two groups based on the median pH of 7.34 (<7.34 vs. ≥ 7.34), using a 10% absolute change in TFdi as the non-inferiority margin.

A linear mixed-effects model was used to evaluate the effect of each therapy on the physiological parameters assessed, with individuals included as a random effect to account for repeated measures. Post hoc pairwise comparisons were performed using estimated marginal means to assess differences between conditions, with p-values adjusted using the Tukey method.

We also conducted a complementary analysis using a Bayesian linear mixed-effects model with a Student-distributed likelihood. The model included a random intercept for each participant and incorporated either informative or neutral prior for all parameters.

## Results

### Patients

Thirty patients were enrolled from February 2019 to March 2024, but only 21 completed the protocol (details in eFigure-2). Of the nine patients who did not complete the protocol, five were discontinued due to poor tolerance of the research settings (2 cases during NIV and 3 cases during HFNC). Two patients were intubated before the protocol began, suggesting a more severe clinical trajectory compared to those who completed it. One patient was discharged early, indicating a faster recovery, and one withdrew voluntarily. Data from 21 patients were available for analysis (age: 70 ± 11 years; 76% male). COPD was the most prevalent comorbidity, affecting 76% of the patients (see Table 1 for details). For the primary endpoint (TFdi), data from 17 patients were analyzed, since four who completed the protocol had missing or unreliable images, which prevented accurate TF measurement. Baseline characteristics for the 17 patients included in the TFdi analysis are detailed in supplemental eTable-2.

**Table 1:** Baseline characteristics of 21 patients completing the protocol.

Variables	Mean ± SD Median (0.25–0.75 IQR)
Age, yr	69.6 ± 11
Male sex, n (%)	16 (76%)
Body Mass Index, kg/m²	24.0 ± 5
Smoking history, n (%)	
Never	1 (5%)
Past	12 (57%)
Current	8 (38%)
Comorbidities, n (%)	
COPD	16 (76%)
Coronary artery disease	12 (57%)
Diabetes	3 (14%)
Chronic heart failure	9 (43%)
Hypertension	11 (52%)
Obstructive Sleep Apnea	8 (38%)
Spirometry (n = 16)	
FEV_1_, % of prediction	34.6 ± 13
FVC, % of prediction	54.7 ± 21
FEV_1_/FVC, %	44.5 ± 16
pH at enrollment*	7.29 (7.26–7.32)
PCO_2_ at enrollment*	68.0 ± 19
Baseline FiO_2,_ (%)	31 ± 8
Admission to enrollment, (days)	1 (0–3)
Baseline oxygen source	
Nasal prong	17 (81%)
Venturi mask	2 (9.5%)
Room air	2 (9.5%)
Respiratory rate, breaths/min	22 (21–25)
Dyspnea, scale (0−10)	3 (2–3)
TFpi / TFdi ratio (n = 17)	0.32(0.22 – 0.60)
End-Expiratory Thickness	
Diaphragm, mm (n = 17)	1.87 ± 0.56
Parasternal Intercostal, mm (n = 17)	2.67 ± 0.73
Transversus Abdominis, mm (n = 16)	3.73 ± 1.16

Abbreviations: COPD = Chronic Obstructive Pulmonary Disease; FEV_1_ = Forced Expiratory Volume in 1 second; FVC = Forced Vital Capacity; PCO_2_ = partial pressure of carbon dioxide; SpO_2_ = peripheral oxygen saturation; FiO_2_ = Fraction of inspired Oxygen; TFdi = diaphragm thickening fraction; TFpi = parasternal intercostal muscle thickening fraction; * = Data from 18 venous blood gas analyses and 3 arterial blood gas analyses.

### Respiratory muscle activity

**Fig. 1:**
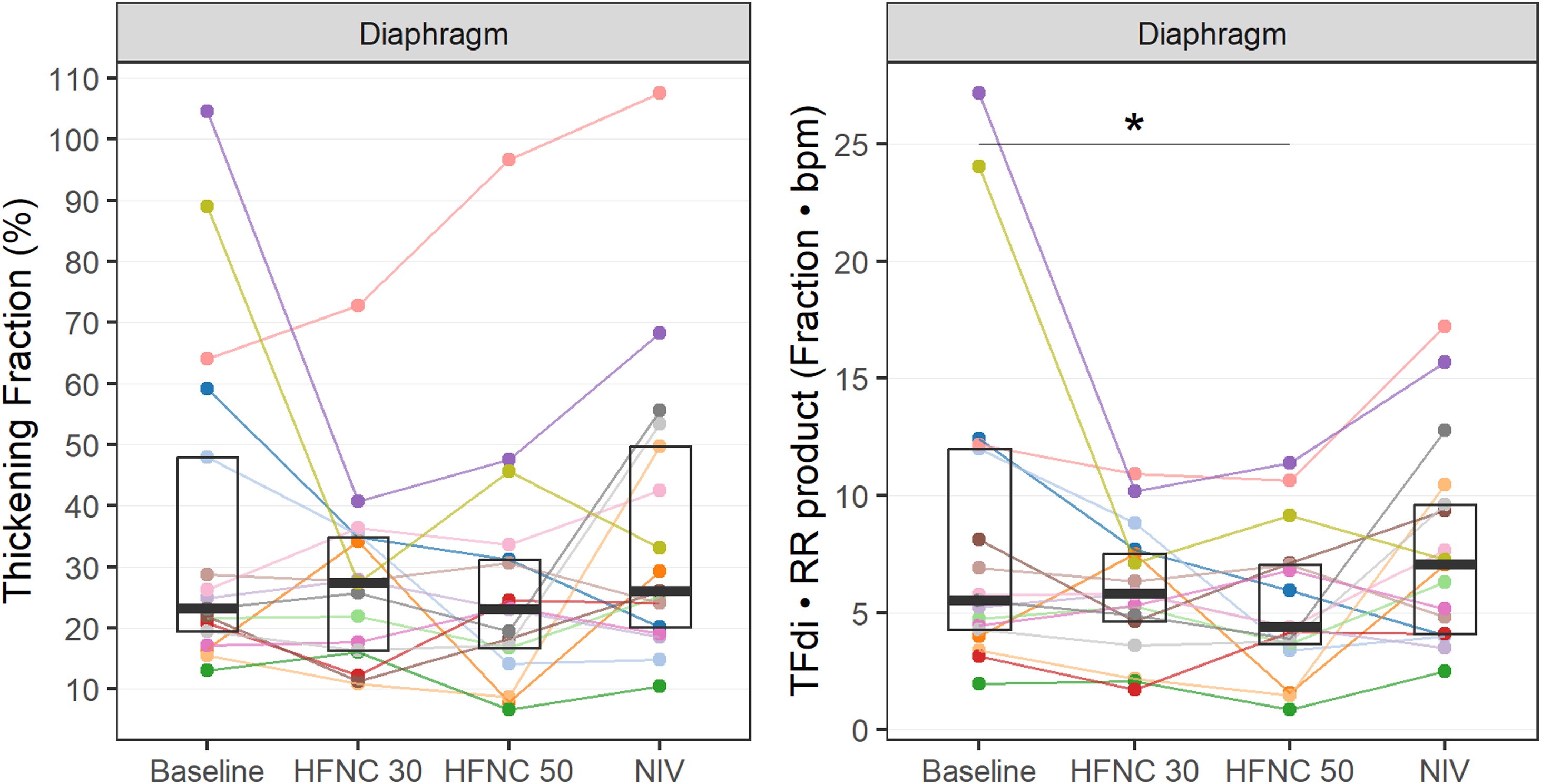
Diaphragm thickening fraction and the product Diaphragm thickening fraction times respiratory rate of the different respiratory muscles during conventional oxygen therapy (baseline), HFNC at 30 L·min^−1^ and 50 L·min^−1^, and during NIV. Each colored line represents individual patient data; boxplots illustrate the distribution of thickening fraction values at each condition, showing the median and interquartile ranges. Abbreviations: HFNC = high flow nasal cannula; NIV = non-invasive ventilation. * = Significant post hoc test.

Physiological outcomes at each protocol step and comparisons are displayed in Table 2 and Fig. 1. HFNC50 was non-inferior to NIV in reducing TFdi, (p = 0.122, 95% CI: -19.1–3.4), based on the pre-specified non-inferiority margin (Supplemental Data - eFig. S3). This non-inferiority finding was further supported by the Bayesian analysis (posterior probability > 0.99; 95% credible interval: –10.97 to 1.32) (Supplemental eTable-3 & eFig. 4a).

Since the median baseline TFdi value was within the normal reference range (23.1%) [[31], [32], [33]], a reduction of 10 percentage points in TFdi was deemed unlikely. Therefore, we also conducted a complementary non-inferiority analysis using a relative 10% reduction in TFdi from baseline. This analysis confirmed the non-inferiority of HFNC50 compared to NIV within a narrowed margin (Wilcoxon, Hodges–Lehmann: p = 0.131, 95% CI: −77.5–9.7) (Supplemental eFigure-4b). Furthermore, the change in TFdi from baseline to NIV showed no meaningful correlation with the level of pressure support applied during NIV (r = 0.16; see Supplemental eFigure-7), suggesting that NIV settings did not directly confound the analysis.

In the exploratory non-inferiority analysis, HFNC30 was found to be non-inferior to NIV when applying an absolute 10-percentage point margin using the Wilcoxon, Hodges–Lehmann methods (p = 0.413; 95% CI: –17.0–5.7). This finding was further supported by the Bayesian analysis (posterior probability >0.99; 95% credible interval: −10.4 to 2.4) (Supplemental eTable-3). However, HFNC30 did not meet the non-inferiority criteria when using a relative 10% change from baseline as the margin (Wilcoxon, Hodges–Lehmann: p = 0.218; 95% CI: –66.4–18.9) (Supplemental eFigure-5).

The TFdi•RR product was significantly reduced during HFNC50 compared to baseline (p = 0.036) (Fig. 1). Moreover, the percentage change in TFdi relative to baseline was significantly reduced during HFNC50 compared to NIV (p = 0.033) (Table 2).

The TFpi and the TFtra remained stable across different ventilatory support modalities (Table 2).

The posterior probability of having smaller values of TFdi during HFNC30 and HFNC50 than during NIV according to the Bayesian mixed-model was 0.84 and 0.90, respectively (Supplemental eTable-3). For TFpi, it was 0.71 and 0.15, and for TFtra, 0.17 and 0.35, respectively (Supplemental eTable-3).

### Ventilation and CO_2_ clearance

**Fig. 2:**
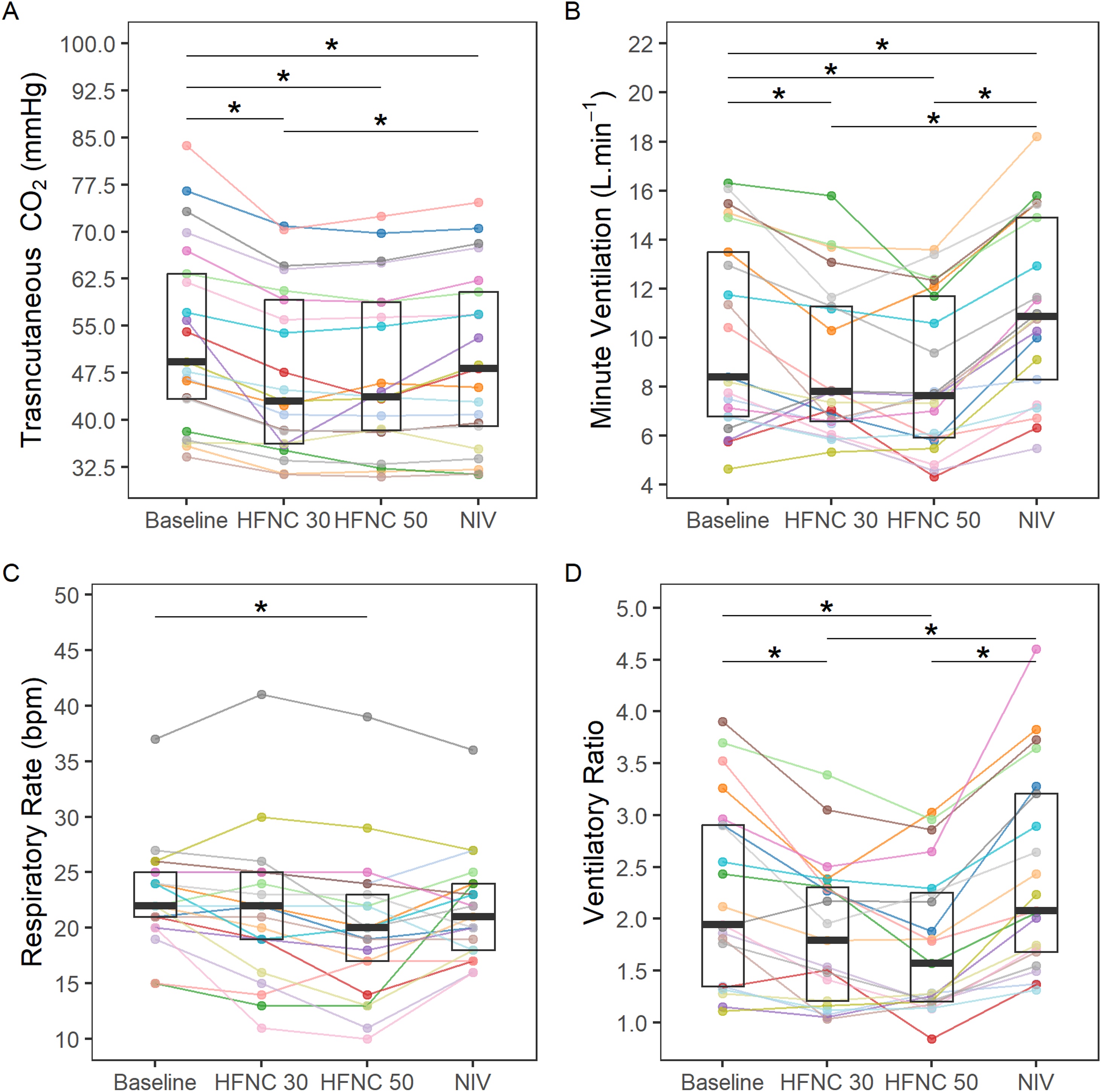
Ventilatory parameters. Each colored line represents individual patient data. Abbreviations: HFNC = High Flow Nasal Cannula; NIV = Non-invasive ventilation * = Significant post hoc test.

Both HFNC50, HFNC30 and NIV led to a significant decrease in tcCO_2_ compared to baseline (Fig. 2a). MV was lower during HFNC compared to either baseline (HFNC30 p = 0.002, and HFNC50 p < 0.001) or NIV (HFNC30 p < 0.001, and HFNC50 p < 0.001) (Fig. 2-b), while NIV increased MV (p = 0.044).

**Fig. 3:**
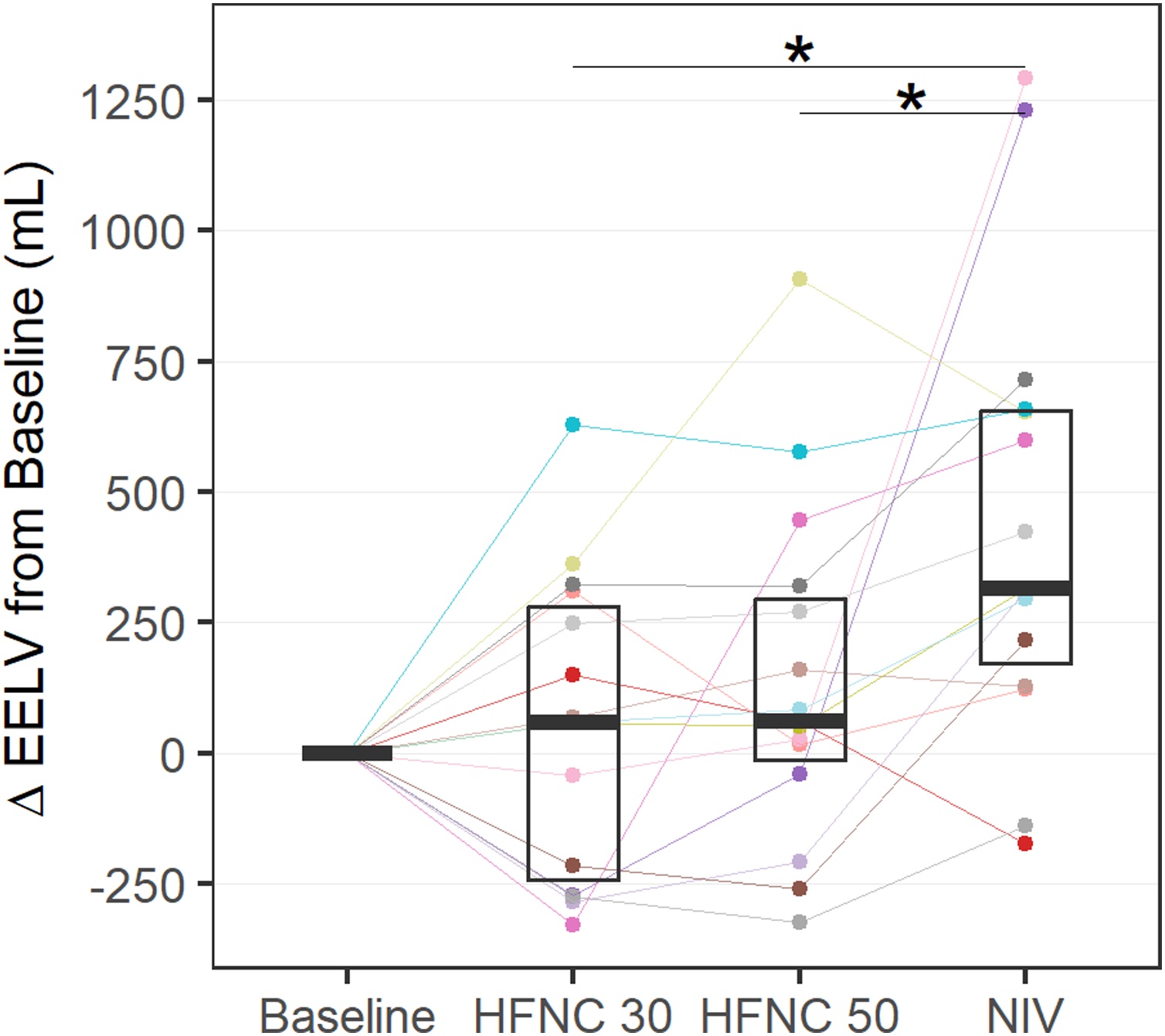
Changes in End-expiratory lung volume during HFNC and NIV. Each colored line represents individual patient data. Abbreviations: HFNC = High Flow Nasal Cannula; NIV = Non-invasive ventilation * = Significant post hoc test.

The decrease in MV from baseline to HFNC50 was primarily driven by a decrease in respiratory rate (p = 0.001), since median tidal volume did not differ across conditions (Fig. 2c, Table 2, and eFigure-7. NIV also reduced tcCO_2_ but with an increase in MV (Fig. 2a,b).

The estimated ventilatory ratio was reduced during HFNC (30 and 50 L.min^−1^) compared to baseline and NIV (Fig. 2b,d), suggesting that HFNC improved ventilation efficiency by reducing physiological dead space.

A Bayesian model estimated the probability of HFNC reducing ventilatory parameters (RR, MV, tcCO_2_, ventilatory ratio, and tidal volume) from baseline and compared to NIV. The probability of tcCO_2_ being lower during HFNC30 and HFNC50 than during NIV was 0.99 and 0.99, respectively. The probability of MV being lower during HFNC30 and HFNC50 than during NIV was >0.99 and >0.99, respectively, and for ventilatory ratio, >0.99 and >0.99, respectively. (Supplemental eTable-3).

### End-expiratory lung volume

In a post hoc analysis, we examined changes in EELV from baseline in the subset of 15 patients with EIT monitoring. A significant increase was observed during NIV compared to both HFNC30 (p = 0.004) and HFNC50 (p = 0.024) (Fig. 3). No change in the ventral–dorsal distribution of ventilation was observed with either therapy, based on the absolute ventral-to-dorsal difference (Table 2).

### Dyspnea and patients’ preference

**Fig. 4:**
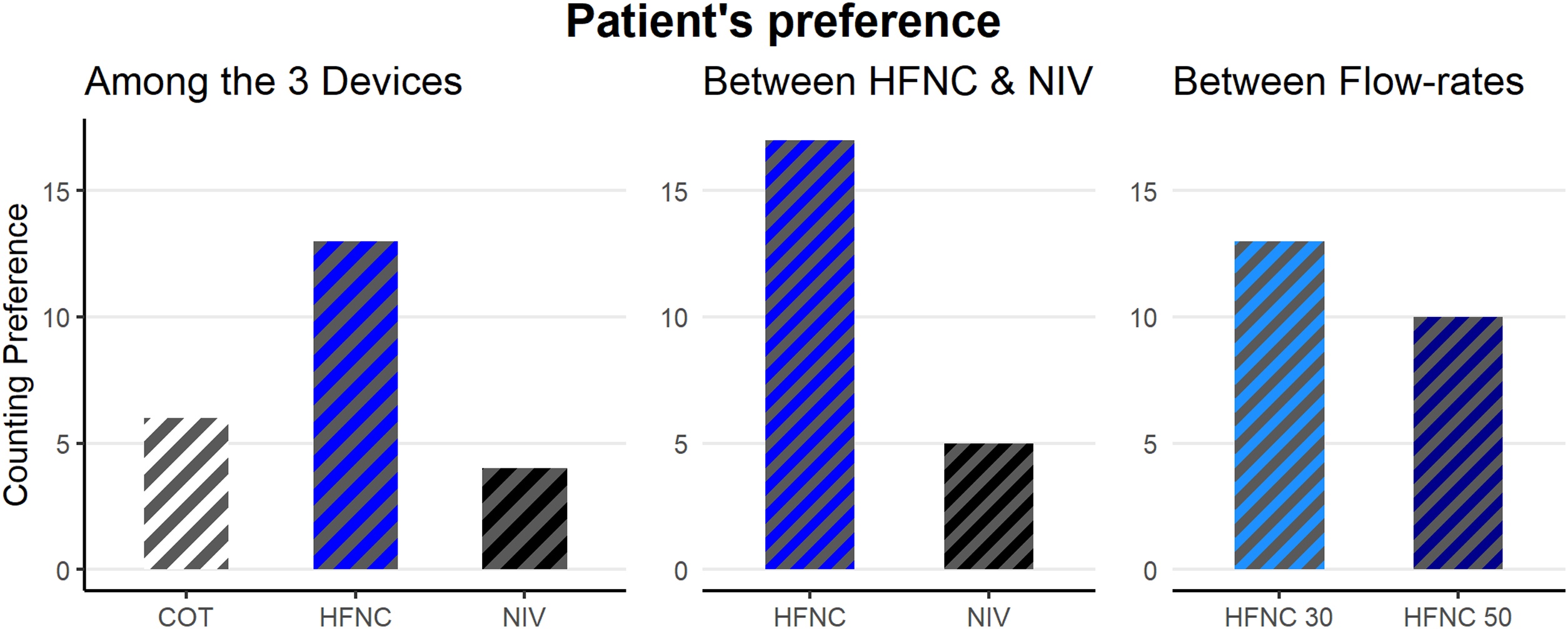
Patient’s preference across devices, and HFNC flow rates. Abbreviations: COT = Conventional Oxygen Therapy; HFNC = High Flow Nasal Cannula; NIV = Non-invasive ventilation. Note: Counted for both if preference was rated equally.

The dyspnea ratings did not differ significantly from baseline or between HFNC and NIV (Table 2). When asked about their preferred device, among COT, HFNC, and NIV, patients most frequently selected HFNC (Fig. 4).

## Discussion

In this crossover, non-inferiority physiological study of patients with stabilized acute hypercapnic respiratory failure following initial stabilization, our main findings were: (1) HFNC50 was non-inferior to NIV in terms of TFdi per breath; (2) only HFNC at 50 L.min^−1^ and not NIV led to a significant reduction in the TFdi•RR product compared to baseline; (3) both HFNC and NIV reduced CO₂ levels; (4) HFNC 30 and 50 L.min^−1^ were associated with reductions in MV and ventilatory ratio compared to baseline, whereas NIV increased MV, suggesting different mechanisms of action; (5) HFNC was preferred by patients over both NIV and COT most of the time.

Trials comparing HFNC and NIV in the management of hypercapnic respiratory failure have shown HFNC to be non-inferior to NIV for gas exchange [18], and prevention of respiratory failure post-extubation [34,35] but meta-analysis of trials have not shown convincing evidence for a difference [23,24]. To illustrate these discrepant results a recent trial on acute respiratory failure found HFNC to be non-inferior to NIV in terms of treatment failure in the subgroup with COPD exacerbation [36]. However, a recent randomized clinical trial in COPD exacerbation showed higher incidence of treatment failure with HFNC compared to NIV [20]. Our initial intent was to focus on COPD. However, patients are often admitted for undifferentiated hypercapnic respiratory failure on top of chronic CO_2_ retention [[3], [4], [5]]. Whether results differ across the different categories would require more attention.

In the present study, HFNC50 was found to be non-inferior to NIV in reducing TFdi, with a non-inferiority margin of absolute 10 percent, also supported by Bayesian analysis. In our investigation, the median baseline TFdi value was within the normal reference range (23.1%) [[31], [32], [33]] making an absolute 10-percentage point reduction unlikely but a complementary analysis using a relative 10% reduction in TFdi from baseline as the non-inferiority margin further supported the non-inferiority of HFNC50.

Although previous studies have shown reduced TFdi during HFNC and NIV compared to COT [37], our different results may be partly explained by differences in baseline TFdi, enrollment timing, and patients’ severity. In our cohort, the median baseline TFdi reflected a normal inspiratory diaphragm effort, whereas in Longhini’s cohort, the effort appeared increased (TFdi 58%), which may have led to a different response to non-invasive support techniques. Recently, Boscolo et al. [38] found that, with baseline TFdi values also in the normal range (28%), HFNC did not reduce absolute TFdi values in patients with acute hypoxemic postextubation respiratory failure, consistent with our data. Normal TFdi under COT may reflect that patients were already stabilized despite our efforts to enroll patients early, but in COPD, it might still hide a higher breathing effort due to the compensatory recruitment of accessory muscles. Hyperinflation places the diaphragm at a mechanical disadvantage and shifts work to extradiaphragmatic muscles, which is supported by an elevated TFpi/TFdi ratio; median of 0.32 in our cohort at baseline (Table 1) versus ∼0.15 in healthy subjects [39].

Rittayamai et al. [26] reported that in hypercapnic COPD patients with mild to moderate exacerbation, HFNC at 30 L.min^−1^ reduced inspiratory effort to a level comparable to NIV, while higher flows showed a trend toward increasing the inspiratory effort in some patients. In contrast, our study demonstrated a better overall physiological response at HFNC50, despite HFNC30 being preferred by patients. Similarly, Colaianni et al. [40] found greater comfort at 30–40 L.min^−1^ with a TFdi comparable to NIV, with higher TFdi at 50–60 L.min^−1^, suggesting increased diaphragmatic workload at higher flow rates. The discrepancy may be explained by the modest EELV increase during HFNC50, particularly as mouth position was not controlled, likely limiting its effect in already stabilized patients and may not reflect the acute phase.

The level of NIV pressure support showed no correlation with TFdi changes, and no overall TFdi reduction was observed during NIV, despite expectations based on prior studies in hypercapnic respiratory failure [27,37]. HFNC50 reduced the TFdi•RR product compared to baseline and achieved a greater percentage decrease in TFdi relative to baseline, whereas NIV did not lower the TFdi•RR product. These findings are consistent with our data in healthy volunteers, showing that reduced respiratory rate is the main factor in lowering overall respiratory workload during HFNC [41]. A meta-analysis in hypercapnic respiratory failure showed HFNC significantly reduced respiratory rate versus NIV [42]. Our previous investigation in healthy volunteers [41] demonstrated that the reduction in work of breathing per minute (i.e. power) during HFNC is mediated by a lower respiratory rate, driven in part by additional expiratory resistance prolonging the expiratory phase. Pinkham et al. [43] further showed that this is accompanied by lower minute ventilation with preserved alveolar ventilation, reflecting improved ventilatory efficiency through upper-airway dead-space washout. This creates a virtuous cycle in which a lower breathing frequency improves CO_2_ clearance, further reducing respiratory rate and the work of breathing.

The lack of TFdi reduction during NIV compared to baseline may be linked to increased EELV, an undesired effect observed in a subset of 15 patients, as it could impact comfort and respiratory effort efficiency. A previous study reported increased EELV with decreased tcCO_2_ during HFNC [44]. While NIV offers effective support, its impact on workload reduction depends on individual comfort and compliance.

Our data showed that both therapies effectively lowered tcCO_2_ within a 15-minute assessment, which aligns with previous trials that reported a CO₂ reduction with both therapies when comparing HFNC and NIV [19,20,24,45]. However, the mechanisms for improving ventilation to reduce tcCO_2_ differed between HFNC and NIV, as HFNC promoted an overall reduction in MV and ventilatory ratio, whereas NIV increased MV and did not improve ventilatory ratio. A previous study reported that HFNC at 40 L.min^−1^ reduced the baseline ventilatory ratio by 18% in patients with hypercapnic respiratory failure [15]; similarly, our data showed a baseline reduction of 21% and 16% during HFNC30 and HFNC50, respectively. Conversely, NIV improves ventilation primarily by increasing tidal volume, leading to CO_2_ clearance, even with the potential for increased dead space from the apparatus [46,47].

Dyspnea perception did not differ from baseline during HFNC or NIV, consistent with previous trials in hypercapnic respiratory failure [20]. However, HFNC was consistently rated superior in comfort [20,35,36], aligning with our findings; patients overall preferred HFNC over NIV. Interestingly, most favored the moderate flow of 30 L.min^−1^ over 50 L.min^−1^, suggesting that individual titration based on both physiological response and comfort may be advisable. This preference supports earlier evidence of COPD patients tolerating flows around 32 L.min^−1^ during the first 24 h [48].

The 2022 European Respiratory Society guidelines recommend a trial of NIV before HFNC in COPD with acute hypercapnic respiratory failure but acknowledge HFNC as an alternative for stabilized acidosis, including for use during NIV breaks, and emphasize the need for more evidence [14]. While NIV has been shown to reduce intubation rates and improve outcomes [[8], [9], [10]], its failure remains a significant concern [11]. Additionally, face mask NIV cannot be used continuously, alternative respiratory support is needed between sessions. This physiological trial demonstrates the benefits of HFNC in this population, providing complementary evidence to support its role as an adjunct or an alternative to NIV.

In patients with active or severe respiratory acidosis (pH < 7.25), NIV remains the first-line therapy; however, HFNC is well-positioned as respiratory support during NIV breaks, as our data demonstrate that it maintains CO_2_ clearance efficiency and reduces the power of breathing (TFdi•RR product). The RENOVATE trial (n = 79 COPD exacerbation) supports this efficacy, suggesting HFNC as a safe option when NIV cooperation is poor and while the patient's trajectory is being established [36]. In stabilized patients or those with mild-to-moderate acidosis, our results more directly apply, supporting HFNC as a primary alternative to NIV, particularly in patients with poor NIV tolerance. This is also supported by Pantazopoulos et al. (n = 105, mild-to-moderate hypercapnic COPD exacerbation), who showed that HFNC achieved comparable CO_2_ clearance to NIV at 1st hour and again from 6 to 72 h [49]. Further clinical trials are needed to establish the optimal role of HFNC in this setting.

### Limitations

The non-invasive ventilatory ratio has not been formally validated against the classical invasive method, even though both EIT and tcCO_2_ have been validated for quantifying ventilation and CO_2_ levels respectively [29,[50], [51], [52]]. NIV settings were not standardized and were applied according to clinical practice, resulting in variability in the level of ventilatory assistance. No correlation was observed between pressure support and changes in TFdi, suggesting that NIV settings did not directly confound the analysis. Patients in this study were often already stabilized after the initial management, exhibiting a normal TFdi, low FiO_2_ requirements, and mild symptoms (reflected by low dyspnea scores) which need to be considered when interpreting our findings. At enrollment, patients were acidotic; however, blood gases were not systematically repeated, and some patients had already improved by the time of data recording. Our protocol did not include instructions regarding mouth position during HFNC, and mouth opening markedly reduces the positive airway pressure generated by HFNC. Prior investigation has shown that mouth closure increases flowdependent nasopharyngeal pressure, prolongs expiration, and reduces respiratory rate and effort [41], effects particularly relevant in COPD. Whether patients in our study spontaneously kept their mouths open, and how this may have influenced our findings, cannot be determined. However, the smaller increase in EELV during HFNC50 compared with NIV suggests that the PEEP generated during HFNC50 was likely lower than the median NIV PEEP of 6 cmH_2_O.

The study was conducted at a single center, limiting generalizability. The 15‑minute observation window per step may limit the detection of additional or delayed differences between conditions that could emerge during longer exposure periods. Furthermore, significant proportion of enrolled patients (30%) did not complete the study protocol, leading to a high post-enrollment exclusion rate primarily from protocol intolerance or uninterpretable ultrasound data. Although the remaining sample met the pre-calculated size, the potential for selection bias cannot be excluded. This may be partly attributed to the characteristics of this population in our center, which admits a substantial proportion of individuals experiencing homelessness or social marginalization [53]. These patients often show low healthcare engagement, poor adherence to hospital treatments, and high comorbidity rates, including mental health and substance abuse, which may affect study retention [54]. Lastly, EELV measurements were performed in only a subset of 15 patients.

## Conclusions

In this study, HFNC50 was non-inferior to NIV in reducing TFdi during the short-term management of stabilized hypercapnic respiratory failure, although TFdi was not significantly reduced by either therapy. HFNC50 led to a reduction in patient’s power of breathing (TFdi•RR product) from baseline, whereas NIV did not. HFNC was preferred by most patients. These findings support HFNC as an alternative to NIV for managing stabilized hypercapnic respiratory failure, either as the initial modality or as an adjunct between NIV sessions.

## Authors’ contributions

LB, FV, IT, TP, AS, MD, DJ and AR designed the work. FV, AS, AR, TP, RC, GC, VP, MK, MS and CS collected the data. FV, AS and MLAS analyzed the data. FV, AS, MD and LB drafted the manuscript, and all authors revised the draft of the manuscript. All authors read and approved the final version of the manuscript. All authors agreed to be accountable for all aspects of the work, ensuring that questions regarding accuracy or integrity of any part are thoroughly investigated and resolved.

## Consent for publication

Not applicable.

## Funding

This research was partially supported by the Canadian Lung Association through the Breathing as One - Boehringer Ingelheim Canada COPD Catalyst Grant. AS was supported by the Canadian Lung Association Fellowship. AR was supported by a Canadian Institutes of Health Research (CIHR) Fellowship (#187900). CS received accommodation funding during his stay in Canada from 'La Fundació de TV3', Spain.

## Ethics approval and consent to participate

This study was reviewed and approved by the Unity Health Toronto Research Ethics Board (REB#16-389, April 12th, 2017) and conducted in accordance with the Declaration of Helsinki. Clinical trial registered with clinicaltrials.gov (NCT03033251, January 26th, 2017).

## We want to thank the following collaborator

Orla Smith, Luana T. Melo, Domenico Grieco, David Mackinnon and Lu Chen.

## Availability of data and materials

The data used in the present study is available from the corresponding author upon reasonable request.

## Declaration of competing interest

LB’s laboratory reports funding grants or equipment from Medtronic, Sentec Inc, Stimit, Cerebra Health and Fisher & Paykel healthcare Inc. LB received fees from Fisher Paykel for a symposium and is PI for a study sponsored by Stimit. FV is currently employed by Fisher & Paykel Healthcare, employment which began after the completion of patient enrollment in the study. RC reports grant from European Respiratory Society and French Intensive Care Society, financial support from Fisher and Paykel Healthcare, Löwenstein medical, and AbbVie outside the submitted work. All authors declared that they have no competing interests related to this work.
