# Supplementary material for: High-flow nasal cannula versus noninvasive ventilation in stabilized hypercapnic exacerbation: a physiological crossover trial
Source: Ann Intensive Care. 2026 May 19;16:100092. doi: 10.1016/j.aicoj.2026.100092 (PMC13233617; doi:10.1016/j.aicoj.2026.100092)
Supplement: Supplementary file 1 [file mmc1.docx]

**Supplemental Digital Content**

**eText-1 - Methods: full description**

This study was approved by the Unity Health Toronto Research Ethics Board (REB No. 16-389) and registered on ClinicalTrials.gov (NCT03033251). This physiological investigation was a prospective, randomized, cross-over, non-inferiority trial comparing HFNC therapy at different flow rates (30 & 50L.min^-1^) and NIV. Informed consent was obtained from the patients or their substitute decision-makers. Enrollment was halted during the COVID-19 pandemic and resumed after the pandemic. In accordance with reporting standards, this study follows the CONSORT 2010 Statement: Extension for Randomized Crossover Trials(1). A completed CONSORT checklist is provided in supplemental eTable 1.

*Patients*

We included adult patients (> 40 years of age to ensure homogeneity in the population) admitted to the Emergency Department, ICU, or medical wards at St. Michael's Hospital, in Toronto, Canada. The investigation initially enrolled only patients with exacerbated COPD (6 patients enrolled in this phase) but was later amended (March 2023) to include those with undifferentiated acute on chronic hypercapnic respiratory failure requiring non-invasive respiratory support (NIV or HFNC) because of the difficulty to have a precise diagnosis on admission. Eligibility criteria included: respiratory acidosis (defined as arterial pH ≤ 7.35 and PCO_2_ ≥ 45 mmHg; or venous pH ≤ 7.34 and PvCO_2_ ≥ 50 mmHg), a respiratory rate (RR) ≥20 breaths/min, and the ability to tolerate spontaneous breathing with conventional oxygen therapy for 15 minutes (if deemed safe by the attending clinician). At the time of data recording, pH had often already improved while waiting for clinical stability to perform the baseline condition of the protocol.

Patients with urgent need for intubation, or morbid obesity (Body Mass Index - BMI >40 kg/m²) were excluded as these conditions could compromise accurate ultrasound and electrical impedance tomography (EIT) assessments. Patients with severe respiratory acidosis (arterial pH <7.25 or venous pH <7.20), a decreased level of consciousness (GCS ≤11), hemodynamic instability needing vasopressors, bronchopleural fistula, or uncooperative behavior were initially excluded but could be included if improved within the subsequent 72 hours.

Demographic and clinical data including age, gender, BMI, prior comorbidities, most recent blood gas analyses, pulmonary function test (when available), main reason for hospital admission, and smoking history were collected at enrollment.

*Study protocol*

Measurements were performed during conventional oxygen therapy (COT) as baseline, followed by a randomized sequence of NIV, and HFNC at two flow rates (30 & 50L.min^-1^) each administered for 15 minutes, similar to a previous study(2) in a standardized clinical setting, ensuring consistency in timing and monitoring across conditions. However, the interfaces and delivery methods differed (face mask for NIV and nasal cannula for HFNC), making blinding not feasible. We performed a two-step randomization using sealed opaque envelopes, first we randomized the ventilatory therapy (HFNC or NIV), followed by the order of HFNC flow rates (30L.min^-1^ or 50L.min^-1^), see eFigure 1. A 5-minute washout period with COT (via nasal cannula or Venturi mask titrated to maintain SpO_2_ within the range specified by the attending physician) was included between NIV and HFNC, as well as between the HFNC flow rates. This duration, which helped reduce the total duration of the protocol, was considered sufficient to minimize carry-over effects since, as only the last 5 minutes of each condition were analyzed, which also helped reduce the total duration of the protocol. During this period, vital signs (i.e., blood pressure, heart rate, and respiratory rate) were collected, and ultrasound images of the respiratory muscles were acquired.

HFNC therapy was delivered using the Bellavista 1000e ventilator (Vyaire Medical Inc, Mettawa, IL, USA), with gas heated and humidified (M850, Fisher & Paykel Healthcare, Auckland, NZ) to 37ºC (or 31ºC if uncomfortable) delivered in accordance with standard practice at the time of protocol conception, using symmetrical, medium-sized nasal cannulas (OPT944, Fisher & Paykel Healthcare) at the baseline FiO_2_.

NIV was also delivered using the Bellavista 1000e ventilator in a dual-limb configuration through a non-vented oronasal mask (FreeMotion RT041, Fisher & Paykel Healthcare, Auckland, NZ). If patients were already receiving NIV, the settings were maintained throughout the study. For all patients, the ventilator settings were initiated according to standard local practice at St. Michael's Hospital for hypercapnic patients initiated on NIV, which consists of starting with a PEEP of 5 cmH_2_O and a pressure support of 8–10 cmH_2_O, with subsequent adjustments made according to clinical judgment (Table 2). For patients already receiving NIV prior to the study, their existing settings were maintained unchanged throughout.

*Ultrasound*

We measured diaphragm thickening fraction (TFdi) as a surrogate of the inspiratory effort(3), and to estimate of the patient’s power of breathing we calculated the TFdi-respiratory rate product (TFdi•RR). This parameter is conceptually analogous to the pressure–time product per minute of inspiratory muscle pressure, reflecting the overall workload of the respiratory muscles. Ultrasound images for thickening fraction of the right diaphragm, parasternal intercostal (TFpi) and transversus abdominis muscles (TFtra) were acquired using a 12-15 MHz linear array transducer (Ultrasound System Vivid e95, General Electric Healthcare, Boston, MA, USA) by one of the two operators, (AS and GC with 10, and 3 years of expertise in the technique, respectively). Muscles were first identified in B-mode, and images were subsequently acquired in M-mode. To ensure consistency across measurements, the transducer placement site was marked on the skin during the first measurement. Measurements were performed offline by the same operators using DICOM Image Viewer software (Horos Project; horosproject.org), corresponding to the therapy administered at the time of analysis. They included inspiratory and expiratory thickness and the computation of thickening fraction for all the above-mentioned respiratory muscles. Details on measurements have been described elsewhere(4-6).

Thickening fraction (TF) within the last 5 minutes of each condition was calculated as the percentage change in muscle thickness between end-expiration and peak inspiration for the inspiratory muscles, and between end-inspiration and peak expiration for the transversus abdominis, reflecting in both cases the percentage change between the most relaxed condition and peak contractile effort of the respective muscle, averaged over 3–5 breaths. These measurements were performed blindly.

Obtaining reliable images (and therefore accurate measurements) of the diaphragm was not possible in 3 patients due to difficult visualization or discrimination of the two layers defining the muscle boundaries, related to extreme diaphragmatic thinning or to poor acoustic window due to hyperinflation.

*Electrical Impedance Tomography (EIT)*

Two noninvasive impedance-based techniques, calibrated against the ventilator, were used to measure tidal volume, respiratory rate and MV, the ExSpiron (ExSpiron 1Xi, Respiratory Motion, Inc., Waltham, MA, USA) in 6 initial patients, and the EIT (PulmoVista 500, Dräger, Lübeck, Germany) in 15 following patients. The EIT also was used to monitor changes in end-expiratory lung volume (EELV) and ventral-to-dorsal ventilation difference (V-D difference)(7). For EIT assessment, a 16-electrode silicone belt was positioned on the chest circumference between the 4^th^ and 5^th^ intercostal spaces and connected to the EIT monitor. EIT calibration enables the conversion of tidal impedance changes into volume as validated in our previous study(8). Prior to the start of the protocol, we calibrated EIT against the tidal volume displayed by the ventilator during NIV, using a series of 20 consecutive breaths. During this process, EIT data were recorded at 50 Hz, alongside the tidal volume of each corresponding breath. To ensure reliable tidal volume measurements, we minimized air leaks, by having an investigator manually hold the oronasal mask in place throughout the calibration recording. EIT data were recorded at 50 Hz and subsequently processed offline using R (version 4.2.2); a low-pass filter (50 bpm) was applied to remove cardiac artifact. Tidal impedance changes were converted to tidal volume using an offline calibration comparing EIT tidal impedance with tidal volume. Volume tracing enabled the computation of tidal volume, respiratory rate, and MV at each study step.

*Transcutaneous CO_2_*

A transcutaneous CO_2_ (tcCO_2_) sensor probe (SenTec Digital Monitoring System - SenTec, Therwil, Switzerland), was attached after appropriate calibration via an ear clip to the patients’ earlobe and data were recorded at 0.25 Hz, to non-invasively estimate arterial CO_2_ levels continuously throughout the study protocol.

Ventilatory ratio was estimated non-invasively by integrating ventilatory parameters from processed EIT data (MV), tcCO_2_, and demographic data. This approach utilizes the classical formula used with invasive methods (mechanical ventilation data and arterial blood gas analysis): [MV (mL.min^-1^) x PaCO_2_ (mm Hg)] / [predicted body weight x 100 (mL.min^-1^) x 37.5 (mmHg)](9). The ventilatory ratio is an indicator of ventilatory inefficiency and dead space.

*Dyspnea and preference*

At the end of each condition, we asked the patient to subjectively rate their level of dyspnea (on a 0-10 scale, where 0 = extremely easy to breathe and 10 = extremely difficult to breathe) (10). At the end of the protocol, we asked patients to indicate their preferred device (in terms of comfort) among COT, HFNC, and NIV, as well as their preference between HFNC and NIV, and their preferred HFNC flow rate.

**Endpoints**

In this cross-over non-inferiority study, the primary endpoint was the absolute diaphragm thickening fraction per breath, looking for a non-inferiority of HFNC50 compared to NIV. Secondary endpoints included comparisons between NIV and HFNC30, as well as between different HFNC flow rates. Additional outcomes assessed were MV, RR, CO_2_ clearance, a surrogate of patient’s power of breathing (TFdi•RR product), TF of parasternal intercostal and transversus abdominis, dyspnea, and preference.

**Sample Size Calculation and Statistical Analysis**

Patient enrollment began in February 2019 with an initial convenience sample of 30 patients. The sample size was revised based on literature published after the study had already begun. We used the trial published by Longhini et al. (30 patients with acute hypercapnic respiratory failure) as the reference for TFdi during HFNC and NIV(11). Assuming a non-inferiority margin of an absolute 10-percentage-point difference in TFdi between HFNC50 and NIV and using a crossover design with a power of 0.90 and a significance level of 0.05, we calculated a sample size of 17 patients. A relatively high drop-out rate (≈20–30%) was anticipated, as hyperinflation (often observed in COPD) can flatten the diaphragm and reduce the zone of apposition, making the assessment of thickness difficult and thickening fraction measurements unreliable in some cases. Additional drop-outs were also expected given the characteristics of the COPD population typically admitted to our center, where social marginalization and comorbidities are common(12).

After assessing normality using the Shapiro–Wilk test, continuous variables were presented as mean and standard deviation (SD) for normally distributed data, or as median and interquartile range (IQR) for non-normally distributed data. Categorical variables were expressed as frequencies and percentages. Non-inferiority test between HFNC50 and NIV was evaluated using the Wilcoxon signed-rank test with Hodges–Lehmann estimates and 95% confidence intervals, applying a non-inferiority margin of absolute 10 percentage points.

A 10% non-inferiority margin was chosen to reflect a difference considered clinically acceptable, supported by methodological considerations. In a previous trial, the reduction in TFdi during HFNC and NIV compared to baseline exceeded 10%(11), suggesting that this threshold captures clinically meaningful changes. Additionally, Vivier’s study reported inter-evaluator variability in TFdi measurements of up to 8%(3). By setting the margin above this variability, we aimed to ensure that observed differences would reflect true physiological effects rather than measurement noise. Therefore, the 10% margin accounts for variability and clinical relevance.

An exploratory non-inferiority analysis was performed comparing HFNC30 with NIV. In addition, a 10% relative change from baseline in TFdi was used as an exploratory non-inferiority margin to compare both HFNC30 and HFNC50 against NIV. As a sensitivity analysis, a subgroup non-inferiority test was conducted between HFNC50 and NIV, stratifying patients by the pH value closest to the time of data recording. Patients were divided into two groups based on the median pH of 7.34 (<7.34 vs. ≥7.34), using a 10% absolute change in TFdi as the non-inferiority margin.

A linear mixed-effects model was used to evaluate the effect of each therapy on the physiological parameters assessed, with individuals included as a random effect to account for repeated measures. Post hoc pairwise comparisons were performed using estimated marginal means to assess differences between conditions, with p-values adjusted using the Tukey method.

To express the results as probabilities and account for the small sample size, also to support the non-inferiority findings, we conducted a complementary analysis using a Bayesian linear mixed-effects model with a Student-distributed likelihood. The model included a random intercept for each participant and incorporated either informative or neutral prior for all parameters. We estimated the model using Markov chain Monte Carlo sampling with four chains, 4000 iterations per chain, and a 2000-sample warm-up period. We ran two different models: 1) the model with specified informative priors for the intercept(9, 13-16), fixed effects, and random effects, and 2) the model using neutral priors.

References: eText-1

1. Dwan K, Li T, Altman DG, Elbourne D. CONSORT 2010 statement: extension to randomised crossover trials. *BMJ* 2019; 366: l4378.

2. Rittayamai N, Phuangchoei P, Tscheikuna J, Praphruetkit N, Brochard L. Effects of high-flow nasal cannula and non-invasive ventilation on inspiratory effort in hypercapnic patients with chronic obstructive pulmonary disease: a preliminary study. *Ann Intensive Care* 2019; 9: 122.

3. Vivier E, Mekontso Dessap A, Dimassi S, Vargas F, Lyazidi A, Thille AW, Brochard L. Diaphragm ultrasonography to estimate the work of breathing during non-invasive ventilation. *Intensive Care Med* 2012; 38: 796-803.

4. Dres M, Dube BP, Goligher E, Vorona S, Demiri S, Morawiec E, Mayaux J, Brochard L, Similowski T, Demoule A. Usefulness of Parasternal Intercostal Muscle Ultrasound during Weaning from Mechanical Ventilation. *Anesthesiology* 2020; 132: 1114-1125.

5. Goligher EC, Fan E, Herridge MS, Murray A, Vorona S, Brace D, Rittayamai N, Lanys A, Tomlinson G, Singh JM, Bolz SS, Rubenfeld GD, Kavanagh BP, Brochard LJ, Ferguson ND. Evolution of Diaphragm Thickness during Mechanical Ventilation. Impact of Inspiratory Effort. *Am J Respir Crit Care Med* 2015; 192: 1080-1088.

6. Schreiber AF, Bertoni M, Coiffard B, Fard S, Wong J, Reid WD, Brochard LJ, Piva S, Goligher EC. Abdominal Muscle Use During Spontaneous Breathing and Cough in Patients Who Are Mechanically Ventilated: A Bi-center Ultrasound Study. *Chest* 2021; 160: 1316-1325.

7. Phoophiboon V, Rodrigues A, Vieira F, Ko M, Madotto F, Schreiber A, Sun N, Sousa MLA, Docci M, Brault C, Menga LS, Telias I, Piraino T, Goligher EC, Brochard L. Ventilation distribution during spontaneous breathing trials predicts liberation from mechanical ventilation: the VISION study. *Crit Care* 2025; 29: 11.

8. Vieira F, Bezerra FS, Coudroy R, Schreiber A, Telias I, Dubo S, Cavalot G, Pereira SM, Piraino T, Brochard LJ. High Flow Nasal Cannula compared to Continuous Positive Airway Pressure: a bench and physiological study. *J Appl Physiol (1985)* 2022; 69.

9. Sinha P, Fauvel NJ, Singh S, Soni N. Ventilatory ratio: a simple bedside measure of ventilation. *Br J Anaesth* 2009; 102: 692-697.

10. Parshall MB, Schwartzstein RM, Adams L, Banzett RB, Manning HL, Bourbeau J, Calverley PM, Gift AG, Harver A, Lareau SC, Mahler DA, Meek PM, O'Donnell DE, American Thoracic Society Committee on D. An official American Thoracic Society statement: update on the mechanisms, assessment, and management of dyspnea. *Am J Respir Crit Care Med* 2012; 185: 435-452.

11. Longhini F, Pisani L, Lungu R, Comellini V, Bruni A, Garofalo E, Laura Vega M, Cammarota G, Nava S, Navalesi P. High-Flow Oxygen Therapy After Noninvasive Ventilation Interruption in Patients Recovering From Hypercapnic Acute Respiratory Failure: A Physiological Crossover Trial. *Crit Care Med* 2019; 47: e506-e511.

12. Lachaud J, Yusuf AA, Maelzer F, Perri M, Gogosis E, Ziegler C, Mejia-Lancheros C, Hwang SW. Social isolation and loneliness among people living with experience of homelessness: a scoping review. *BMC Public Health* 2024; 24: 2515.

13. Fraser JF, Spooner AJ, Dunster KR, Anstey CM, Corley A. Nasal high flow oxygen therapy in patients with COPD reduces respiratory rate and tissue carbon dioxide while increasing tidal and end-expiratory lung volumes: a randomised crossover trial. *Thorax* 2016; 71: 759-761.

14. Piquilloud L, Olivier PY, Richard JC, Thepot-Seegers V, Brochard L, Mercat A, Beloncle F. High flow nasal cannula improves breathing efficiency and ventilatory ratio in COPD patients recovering from an exacerbation. *J Crit Care* 2022; 69: 154023.

15. Rittayamai N, Marinpong V, Chuaychoo B, Tscheikuna J, Brochard LJ. Ultrasound Evaluation of Parasternal Intercostal, Diaphragm Activity, and Their Ratio in Male Patients with Chronic Obstructive Pulmonary Disease. *Am J Respir Crit Care Med* 2024; 209: 1016-1018.

16. Topcuoglu C, Yumin ET, Hizal M, Konuk S. Examination of diaphragm thickness, mobility and thickening fraction in individuals with COPD of different severity. *Turk J Med Sci* 2022; 52: 1288-1298.

**eTable-1 -** CONSORT Checklist for Randomized Crossover Trial

| Section/Topic | Item No | Checklist item | Reported on page No |
| --- | --- | --- | --- |
| Title and abstract | | | |
|  | 1a | Identification as a randomized trial in the title | 1 |
|  | 1b | Structured summary of trial design, methods, results, and conclusions (for specific guidance see CONSORT for abstracts) | 3 |
| Introduction | | | |
| Background and objectives | 2a | Scientific background and explanation of rationale | 5 – 6 |
|  | 2b | Specific objectives or hypotheses | 6 |
| Methods | | | |
| Trial design | 3a | Description of trial design (such as parallel, factorial) including allocation ratio | 6 & Suppl.1 |
|  | 3b | Important changes to methods after trial commencement (such as eligibility criteria), with reasons | 6 – 7 & Suppl. 1 |
| Participants | 4a | Eligibility criteria for participants | 7 & Suppl. 1-2 |
|  | 4b | Settings and locations where the data were collected | 6 & Suppl. 1 |
| Interventions | 5 | The interventions for each group with sufficient details to allow replication, including how and when they were actually administered | 7 – 10 &  Suppl. 2-6 |
| Outcomes | 6a | Completely defined pre-specified primary and secondary outcome measures, including how and when they were assessed | 10 & Suppl. 6 |
|  | 6b | Any changes to trial outcomes after the trial commenced, with reasons | 6, & Suppl. 1, 6 |
| Sample size | 7a | How sample size was determined | & Suppl. 6-7 |
|  | 7b | When applicable, explanation of any interim analyses and stopping guidelines | NA |
| Randomisation: |  |  |  |
| Sequence generation | 8a | Method used to generate the random allocation sequence | 7 & Suppl. 3 |
|  | 8b | Type of randomisation; details of any restriction (such as blocking and block size) | 7 & Suppl. 3 |
| Allocation concealment mechanism | 9 | Mechanism used to implement the random allocation sequence (such as sequentially numbered containers), describing any steps taken to conceal the sequence until interventions were assigned | 7 & Suppl. 3 |
| Implementation | 10 | Who generated the random allocation sequence, who enrolled participants, and who assigned participants to interventions | 7 & Suppl. 3 |
| Blinding | 11a | If done, who was blinded after assignment to interventions (for example, participants, care providers, those assessing outcomes) and how | 7 & Suppl. 3 |
|  | 11b | If relevant, description of the similarity of interventions | 7 & Suppl. 3 |
| Statistical methods | 12a | Statistical methods used to compare groups for primary and secondary outcomes | 11 – 12  & Suppl. 6 - 8 |
|  | 12b | Methods for additional analyses, such as subgroup analyses and adjusted analyses | 11 – 12  & Suppl. 6 - 8 |
| Results | | | |
| Participant flow (a diagram is strongly recommended) | 13a | For each group, the numbers of participants who were randomly assigned, received intended treatment, and were analysed for the primary outcome | 12  & Suppl. 20 |
|  | 13b | For each group, losses and exclusions after randomisation, together with reasons | 12  & Suppl. 20 |
| Recruitment | 14a | Dates defining the periods of recruitment and follow-up | 12 |
|  | 14b | Why the trial ended or was stopped | 12 |
| Baseline data | 15 | A table showing baseline demographic and clinical characteristics for each group | 12  & Suppl. 14 |
| Numbers analysed | 16 | For each group, number of participants (denominator) included in each analysis and whether the analysis was by original assigned groups | 12  & Suppl. 20 |
| Outcomes and estimation | 17a | For each primary and secondary outcome, results for each group, and the estimated effect size and its precision (such as 95% confidence interval) | 12-15  & Suppl. 21-25 |
|  | 17b | For binary outcomes, presentation of both absolute and relative effect sizes is recommended | 12  & Suppl. 14 |
| Ancillary analyses | 18 | Results of any other analyses performed, including subgroup analyses and adjusted analyses, distinguishing pre-specified from exploratory | 12-15  & Suppl. 21-25 |
| Harms | 19 | All important harms or unintended effects in each group (for specific guidance see CONSORT for harms) | 11  & Suppl. 20 |
| Discussion | | | |
| Limitations | 20 | Trial limitations, addressing sources of potential bias, imprecision, and, if relevant, multiplicity of analyses | 20-21 |
| Generalisability | 21 | Generalisability (external validity, applicability) of the trial findings | 20 |
| Interpretation | 22 | Interpretation consistent with results, balancing benefits and harms, and considering other relevant evidence | 20-21 |
| Other information | | |  |
| Registration | 23 | Registration number and name of trial registry | 6 & Suppl. 1 |
| Protocol | 24 | Where the full trial protocol can be accessed, if available | 6 & Suppl. 1 |
| Funding | 25 | Sources of funding and other support (such as supply of drugs), role of funders | 24-25 |

**eTable-2** - Baseline characteristics: subset of 17 patients included for the analysis of Diaphragm Thickening Fraction

| **Variables** | **Mean±SD or**  **Median (0.25-0.75)** |
| --- | --- |
| Age, yr | 69.3 ± 11 |
| Male sex, n (%) | 12 (71%) |
| Body Mass Index | 24.0 ± 4 |
| Cumulative smoking, n (%)  Never  Past  Current | 1 (6%)  9 (53%)  7 (41%) |
| Comorbidities, n (%)  COPD  Coronary artery disease  Diabetes  Chronic heart failure  Hypertension  Obstructive Sleep Apnea | 14 (82%)  10 (59%)  3 (18%)  9 (53%)  10 (59%)  6 (35%) |
| Spirometry  FEV1, % of prediction (n= 14)  FVC, % of prediction (n= 14)  FEV1/FVC, % (n= 14) | 33 ± 11  59.5 ± 16  43 ± 20 |
| pH at enrollment* | 7.29 (7.26 – 7.32) |
| PCO_2_ at enrollment* | 69.5 ± 19 |
| SpO_2_, % | 94.4 ± 4 |
| Baseline FiO_2,_ % | 31 ± 8 |
| Baseline oxygen source  Nasal prong  Venturi mask  Room air | 13 (76%)  2 (12%)  2 (12%) |
| Respiratory rate, breaths/min | 22 (21 – 25) |
| Dyspnea, scale 0-10 | 3 (2 – 4) |
| End-Expiratory Thickness  Diaphragm, mm (n= 17)  Parasternal Intercostal, mm (n= 17)  Transversus Abdominis, mm (n= 16) | 1.87 ± 0.56  2.67 ± 0.73  3.73 ± 1.16 |

Abbreviations: COPD= Chronic Obstructive Pulmonary Disease; FEV1= Forced Expiratory Volume in 1 second; FVC= Forced Vital Capacity; PCO_2_= partial pressure of carbon dioxide; SpO_2_= peripheral oxygen saturation; FiO2= Fraction of inspired Oxygen; * Data from 15 venous blood gas analyses and 2 arterial blood gas analyses.

**eTable-3 -** Bayesian model with prior & neutral probabilities

| **Variable** | **Prior (Mean±sd)** | **Intercept (CI)** | **Hypothesis** | **Mean difference (CI)** | **Post-probability** |
| --- | --- | --- | --- | --- | --- |
| Thickening Fraction: Diaphragm, (%) | 35.2±6.57  (1) | 30.31  (22.8 - 38.7) | HFNC 30 non-inferior to NIV | -13.81(-20.4 - 7.57) | > 0.99 |
|  |  |  | HFNC 50 non-inferior to NIV | -14.67(-21 - -8.7) | >0.99 |
|  |  |  | HFNC 30 < Baseline | -1.3(-6.43 - 3.41) | 0.66 |
|  |  |  | HFNC 50 < Baseline | -2.16(-7.09 2.53) | 0.78 |
|  |  |  | NIV < Baseline | 2.51(-2.48 - 7.57) | 0.21 |
|  |  |  | HFNC 30 < NIV | -3.81(-10.38 - 2.43) | 0.84 |
|  |  |  | HFNC 50 < NIV | -4.67(-10.97 - 1.32) | 0.9 |
|  |  |  | HFNC 30 < HFNC 50 | 0.86(-4.63 - 6.33) | 0.39 |
| Thickening Fraction: Parasternal Intercostal, (%) | Neutral  Default  11±8.2 | 13.0  (9.2 - 16.8) | HFNC30 < Baseline | -1.66(-4.6 - 1.21) | 0.83 |
|  |  |  | HFNC50 < Baseline | 1.38(-1.47 - 4.2) | 0.22 |
|  |  |  | NIV < Baseline | -0.59(-3.48 - 2.3) | 0.63 |
|  |  |  | HFNC30 < NIV | -1.07(-4.21 - 2.1) | 0.71 |
|  |  |  | HFNC50 < NIV | 1.97(-1.1 - 5.08) | 0.15 |
|  |  |  | HFNC30 < HFNC 50 | -3.04(-6.21 - 0.04) | 0.95 |
| Thickening Fraction: Transversus Abdominis, (%) | Neutral  Default  16.5±10 | 18.52  (14.1 - 23) | HFNC30 < Baseline | 1.3(-3.0 - 5.59) | 0.31 |
|  |  |  | HFNC50 < Baseline | -0.24(-4.5 - 4.1) | 0.54 |
|  |  |  | NIV< Baseline | -1.36(-5.71 - 2.96) | 0.69 |
|  |  |  | HFNC30 < NIV | 2.66(-2.21 - 7.5) | 0.17 |
|  |  |  | HFNC50 < NIV | 1.12(-3.64 - 5.9) | 0.35 |
|  |  |  | HFNC30 < HFNC 50 | 1.54(-3.19 - 6.33) | 0.29 |
| Transcutaneous CO_2_, mmHg | 46.7±9.4  (2) | 52.76  (46.86 - 58.5) | HFNC 30 < Baseline | -3.08(-3.99 - -2.19) | > 0.99 |
|  |  |  | HFNC 50 < Baseline | -3.19(-4.1 - -2.33) | > 0.99 |
|  |  |  | NIV < Baseline | -1.88(-2.79 - 1.01) | >0.99 |
|  |  |  | HFNC 30 < NIV | -1.2(-2.08 - -0.35) | 0.99 |
|  |  |  | HFNC 50 < NIV | -1.3(-2.17 - 0.46) | 0.99 |
|  |  |  | HFNC 30 < HFNC 50 | 0.11(-0.74 - 0.96) | 0.42 |
| Minute Ventilation, L.min^-1^ | 6.5±2.5  (2) | 9.88  (8.4 - 11.3) | HFNC 30 < Baseline | -1.13(-1.76 - -0.49) | >0.99 |
|  |  |  | HFNC 50 < Baseline | -1.64(-2.25 - -1.02) | >0.99 |
|  |  |  | NIV < Baseline | 1.03(0.39 - 1.66) | <0.01 |
|  |  |  | HFNC 30 < NIV | -2.16(-2.79 - -1.53) | > 0.99 |
|  |  |  | HFNC 50 < NIV | -2.67(-3.29 - -2.05) | >0.99 |
|  |  |  | HFNC 30 < HFNC 50 | 0.51(-0.1 - 1.12) | 0.09 |
| Respiratory Rate, bpm | 25.3±3  (3) | 23.03  (20.6 - 25.4) | HFNC 30 < Baseline | -0.91(-2.03 - 0.16 | 0.92 |
|  |  |  | HFNC 50 < Baseline | -2.41(-3.5 - -1.31) | > 0.99 |
|  |  |  | NIV < Baseline | -0.94(-2.03 - 0.19) | 0.92 |
|  |  |  | HFNC 30 < NIV | 0.03(-1.15 - 1.19) | 0.48 |
|  |  |  | HFNC 50 < NIV | -1.47(-2.64 - -0.32 | 0.98 |
|  |  |  | HFNC 30 < HFNC 50 | 1.50(0.35 - 2.63 | 0.02 |
| Tidal Volume, mL | 400± 89  (2) | 449  (370 - 527) | HFNC 30 < Baseline | -19.4(-59.3 - 21) | 0.79 |
|  |  |  | HFNC 50 < Baseline | -6.37(-46.5 - 34.1) | 0.61 |
|  |  |  | NIV < Baseline | 66(22.6 - 107) | 0.01 |
|  |  |  | HFNC 30 < NIV | -85.9(-130 - -39.4) | > 0.99 |
|  |  |  | HFNC 50 < NIV | -73(-115 - -28.5) | >0.99 |
|  |  |  | HFNC 30 < HFNC 50 | -13(-54.8 - 30.5) | 0.69 |
| Ventilatory Ratio | 2.13±1.05  (4) | 2.22  (1.88 - 2.56) | HFNC 30 < Baseline | -0.36(-0.53 - -0.2) | > 0.99 |
|  |  |  | HFNC 50 < Baseline | -0.46(-0.62 - -0.3) | > 0.99 |
|  |  |  | NIV < Baseline | 0.15(-0.03 - 0.32) | 0.08 |
|  |  |  | HFNC 30 < NIV | -0.51(-0.68 - 0.33) | >0.99 |
|  |  |  | HFNC 50 < NIV | -0.61(-0.78 - -0.44) | >0.99 |
|  |  |  | HFNC 30 < HFNC 50 | 0.1(-0.06 - 0.25) | 0.15 |
| TFic/TFdi ratio | 0.52± 0.18  (5) | 0.48  (0.13 - 0.34) | HFNC 30 < Baseline | -0.02(-.013 - 0.08) | 0.64 |
|  |  |  | HFNC 50 < Baseline | -0.08(-0.03 - 0.2) | 0.12 |
|  |  |  | NIV < Baseline | 0.07(-0.11 - 1.01) | 0.5 |
|  |  |  | HFNC 30 < NIV | -0.02(-0.15 - 0.11) | 0.61 |
|  |  |  | HFNC 50 < NIV | 0.08(-0.07 - 0.24) | 0.18 |
|  |  |  | HFNC 30 < HFNC 50 | -0.1(-0.24 - 0.03) | 0.9 |
| Tfdi • RR product,  % • bpm | Neutral Default 565.7±286 | 811  (589 –1024) | HFNC 30 < Baseline | -203.8(-377 - - 23.4) | 0.97 |
|  |  |  | HFNC 50 < Baseline | -259.7(-432 - -79) | 0.99 |
|  |  |  | NIV < Baseline | -33.9(-208 - 144) | 0.63 |
|  |  |  | HFNC 30 < NIV | -169.9 (-357 - 17.6) | 0.93 |
|  |  |  | HFNC 50 < NIV | -225.8 (-414 - -32.1) | 0.97 |
|  |  |  | HFNC 30 < HFNC 50 | 56(-132 - 247) | 0.32 |

Abbreviations: HFNC= High Flow Nasal Cannula; NIV = Non-invasive Ventilation, RR= Respiratory Rate; TFdi= Thickening Fraction of the diaphragm; TFpi= Thickening Fraction of the parasternal intercostal; TFtra= Thickening Fraction of the transversus abdominis; sd= Standard deviation, CI= Credible Interval.

**Reference for priors in the Bayesian Analysis**

1. Topcuoglu C, Yumin ET, Hizal M, Konuk S. Examination of diaphragm thickness, mobility and thickening fraction in individuals with COPD of different severity. Turk J Med Sci. 2022;52(4):1288-98.

2. Fraser JF, Spooner AJ, Dunster KR, Anstey CM, Corley A. Nasal high flow oxygen therapy in patients with COPD reduces respiratory rate and tissue carbon dioxide while increasing tidal and end-expiratory lung volumes: a randomised crossover trial. Thorax. 2016;71(8):759-61.

3. Piquilloud L, Olivier PY, Richard JC, Thepot-Seegers V, Brochard L, Mercat A, et al. High flow nasal cannula improves breathing efficiency and ventilatory ratio in COPD patients recovering from an exacerbation. J Crit Care. 2022;69:154023.

4. Sinha P, Fauvel NJ, Singh S, Soni N. Ventilatory ratio: a simple bedside measure of ventilation. Br J Anaesth. 2009;102(5):692-7.

5. Rittayamai N, Marinpong V, Chuaychoo B, Tscheikuna J, Brochard LJ. Ultrasound Evaluation of Parasternal Intercostal, Diaphragm Activity, and Their Ratio in Male Patients with Chronic Obstructive Pulmonary Disease. Am J Respir Crit Care Med. 2024;209(8):1016-8.


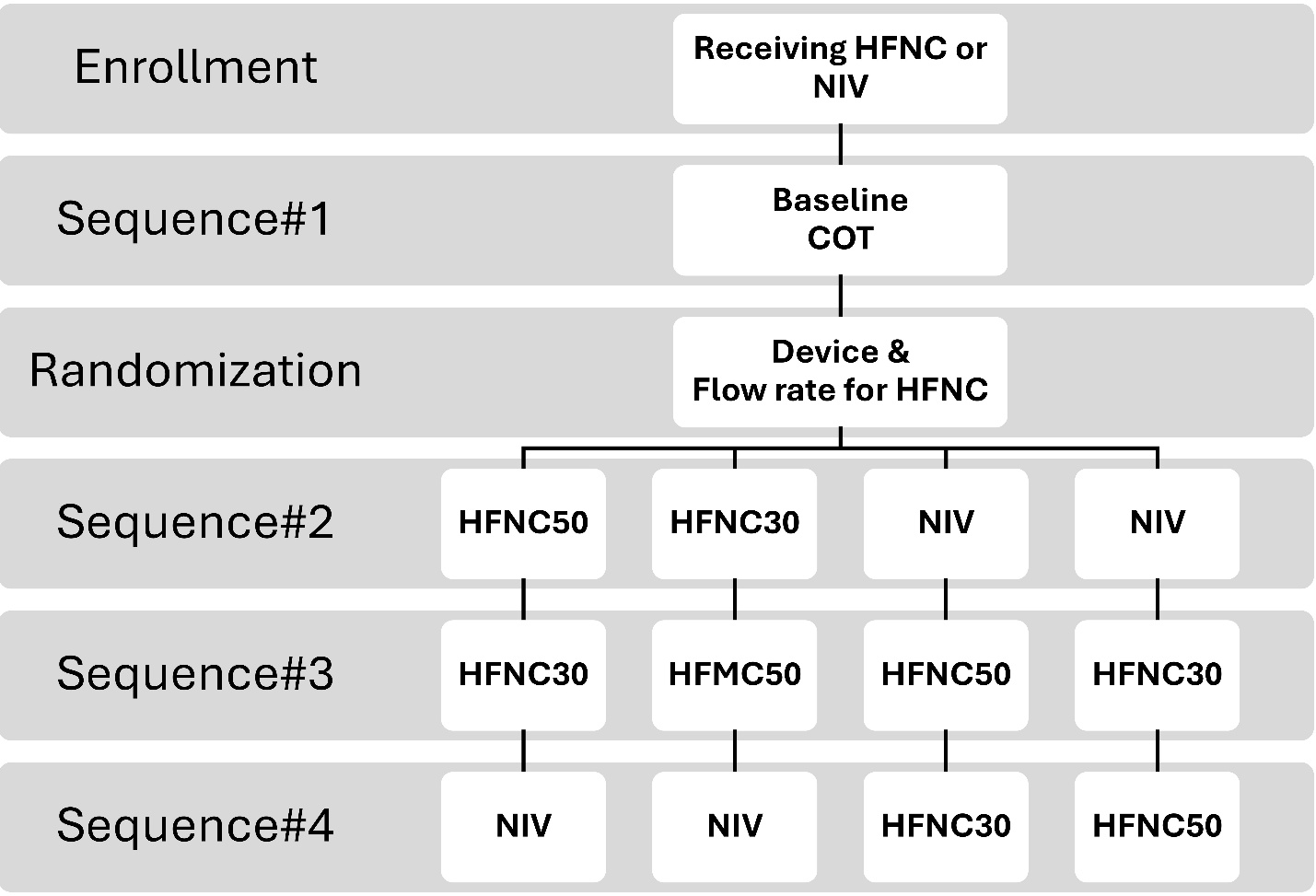


**eFigure-1** – Study Protocol diagram: Overview of the study protocol


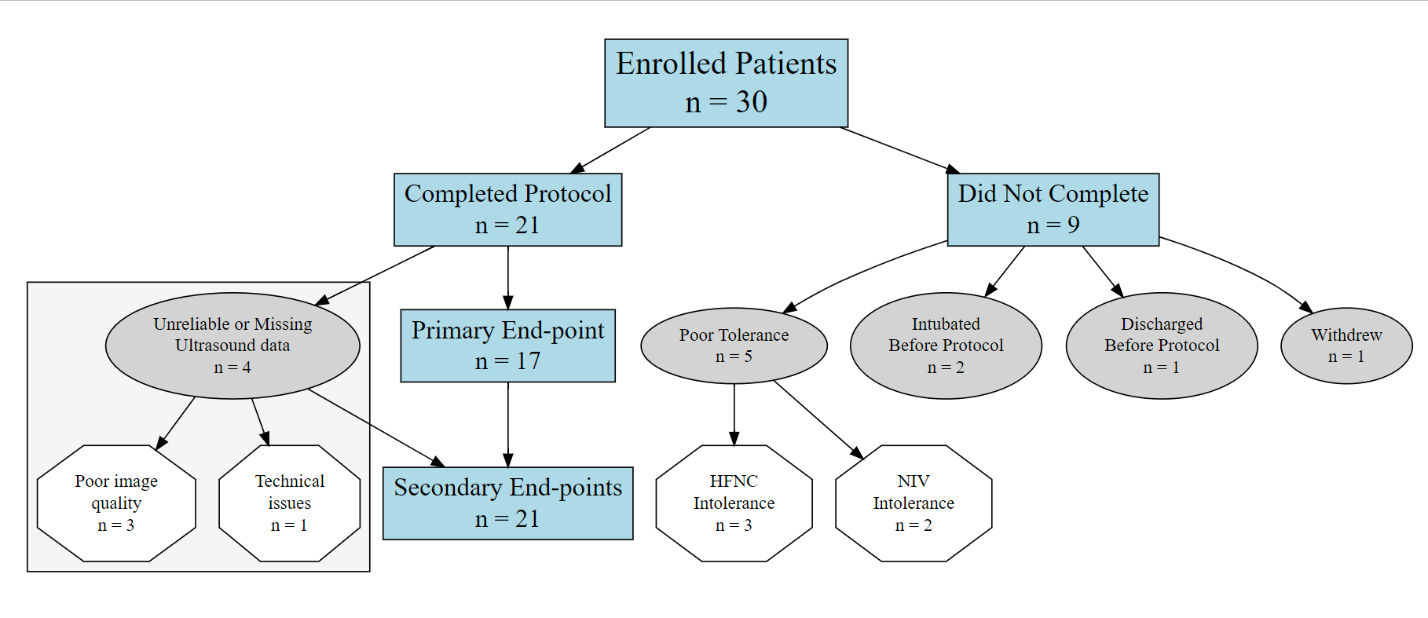


eFigure-2 – Flow diagram: Overview of enrollment, exclusion, and endpoints analysis


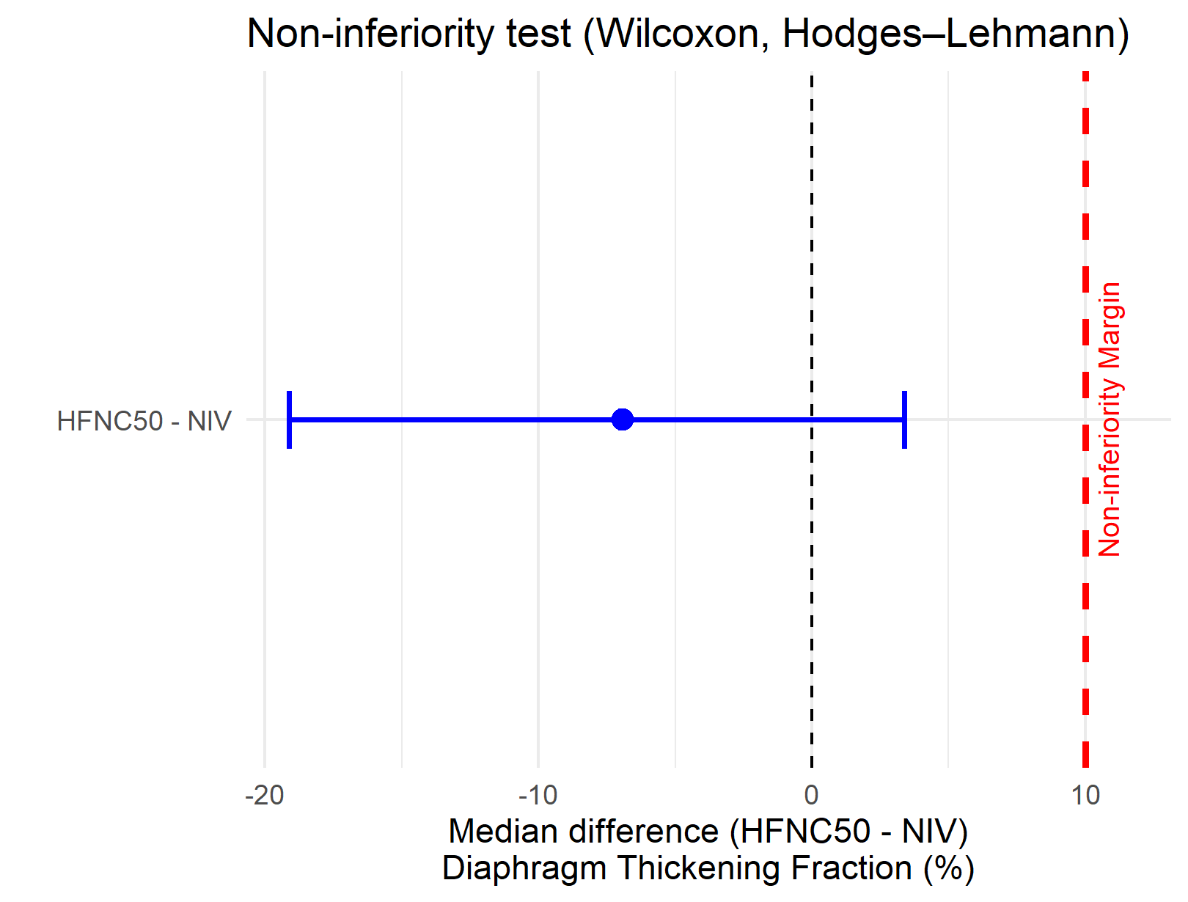


**eFigure-3** – Primary Endpoint: Non-inferiority test comparing HFNC at 50 L.min^-1^ versus NIV, using a 10% absolute diaphragmatic thickening fraction (TFdi) as the non-inferiority margin. HFNC50 was non-inferior to NIV in reducing TFdi, (p = 0.122, 95% CI: -19.1 – 3.4)


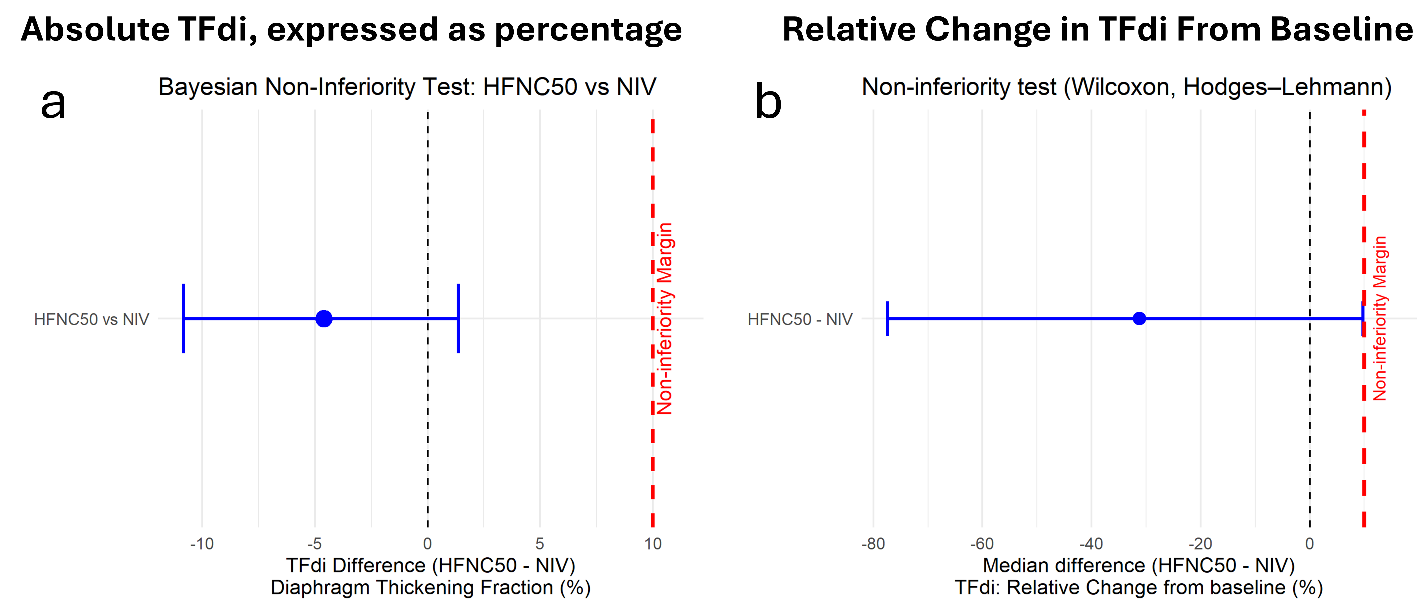


**eFigure-4** - Complementary Non-inferiority test comparing HFNC at 50 L.min^-1^ versus NIV. **a)** Baeysian Non-inferiority test using a 10% absolute TFdi as the non-inferiority margin; HFNC50 was non-inferior to NIV in reducing TFdi, (Posterior probability > 0.99%, 95% Credible Interval: -10.97 – 1.32). **b)** Wilcoxon, Hodges-Lehmann test using 10% relative change from the Baseline as the non-inferiority margin; HFNC50 was non-inferior to NIV in reducing TFdi (p= 0.131, 95% CI: -77.5 – 9.7)


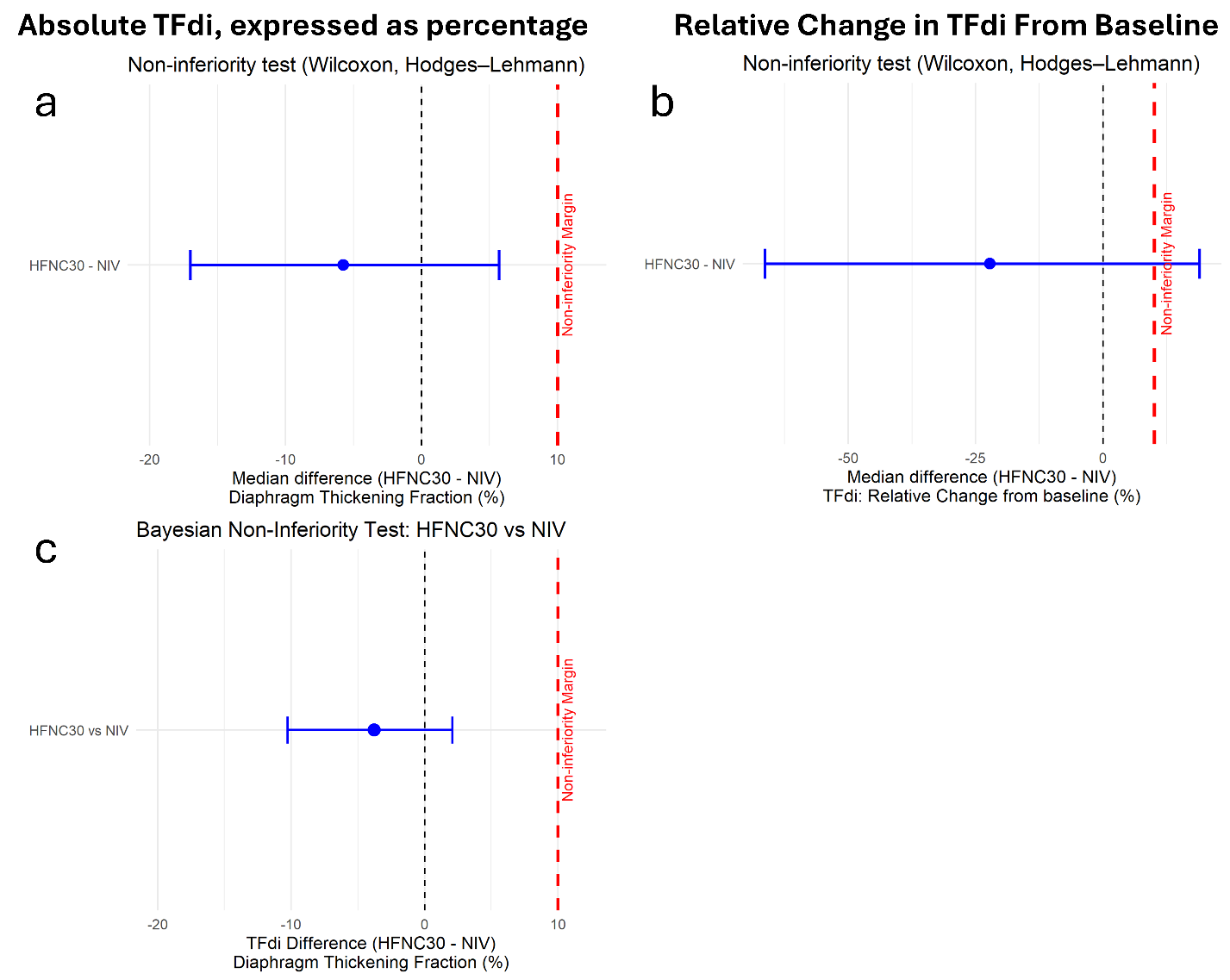


**eFigure-5** – Exploratory analysis: Non-inferiority test comparing HFNC at 30 L.min^-1^ versus NIV. **a)** Wilcoxon, Hodges-Lehmann test using a 10% absolute diaphragmatic thickening fraction (TFdi) as the non-inferiority margin; HFNC30 was non-inferior to NIV in reducing TFdi, (p = 0.413, 95% CI: -17.0 – 5.7). **b)** Wilcoxon, Hodges-Lehmann test using 10% relative change from the Baseline as the non-inferiority margin; HFNC30 did reached the criteria to be non-inferior to NIV in reducing TFdi (p= 0.218, 95% CI: -66.4 – 18.9). **c)** Baeysian non-inferiority test using a 10% absolute TFdi as the non-inferiority margin; HFNC30 was non-inferior to NIV in reducing TFdi, (Posterior probability > 0.99%, 95% Credible Interval: -10.4 – 2 .4).


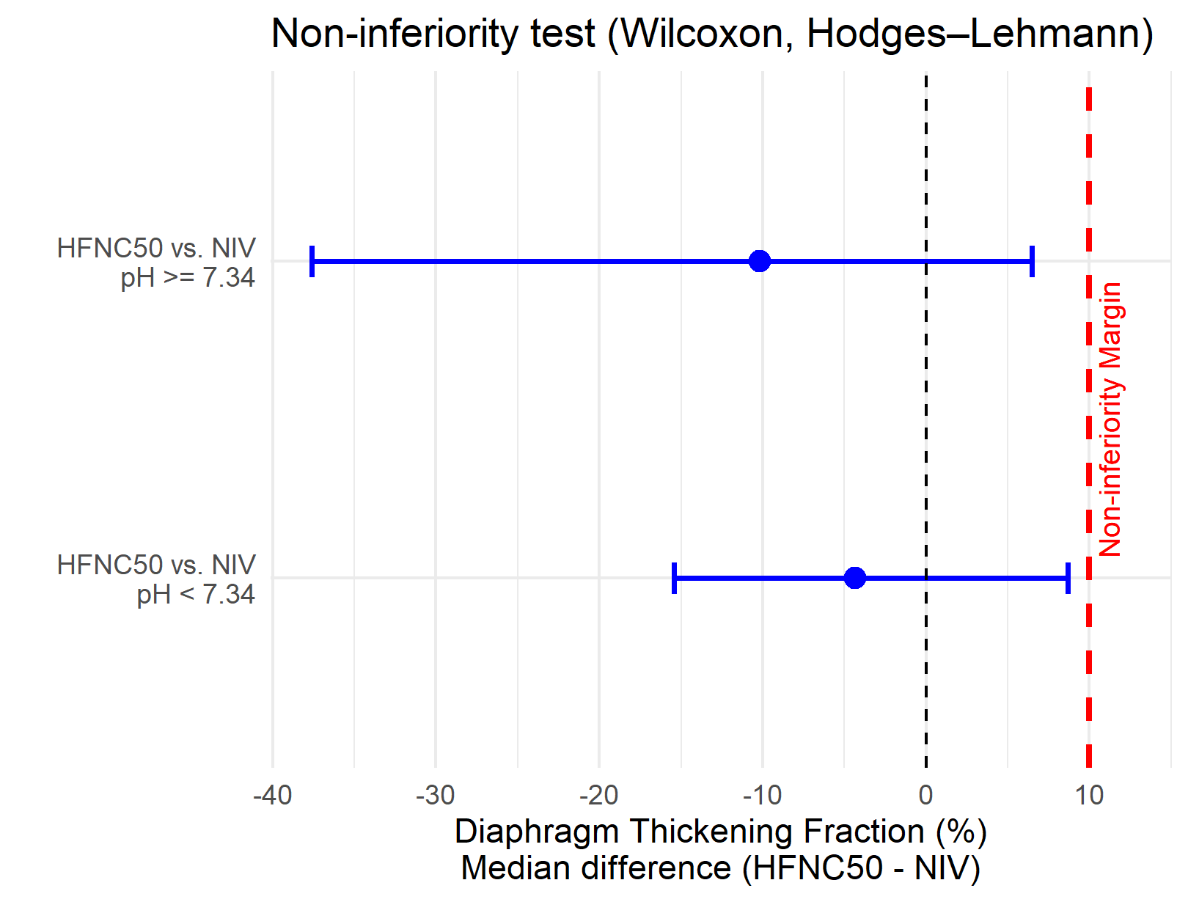


**eFigure-6** – Sensitivity analysis: Non-inferiority test comparing HFNC at 50 L.min^-1^ versus NIV. Wilcoxon, Hodges-Lehmann test using a 10% absolute diaphragmatic thickening fraction (TFdi) as the non-inferiority margin. HFNC50 was non-inferior to NIV in reducing TFdi across both patient groups, stratified by severity of pH acidosis closest to the time of data recording: pH ≥ 7.34 (p = 0.130, 95% CI: -37.6 – 6.5), and pH < 7.34 (p = 0.489, 95% CI: -15.4 – 8.7).


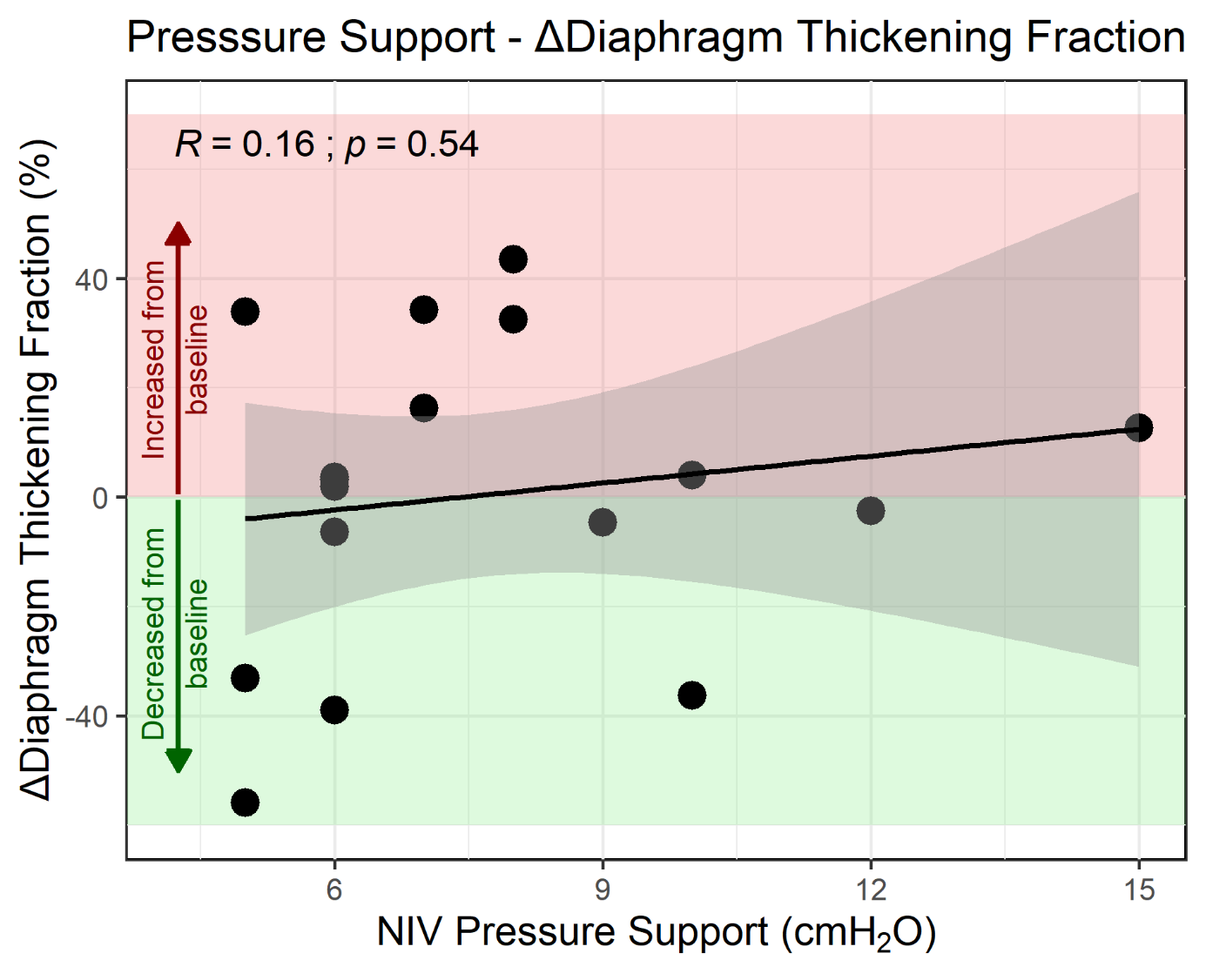


**eFigure-7 –** Correlation between NIV pressure‑support levels and changes in diaphragm thickening fraction from baseline to the NIV condition.
